# Supplementary material for: Morphology and intervesicle distances in condensates of synaptic vesicles and synapsin
Source: Biophys J. 2024 Nov 8;123(23):4123–34. doi: 10.1016/j.bpj.2024.11.004 (PMC11628805; doi:10.1016/j.bpj.2024.11.004)
Supplement: Document S1. Figures S1–S4 and Tables S1–S6 [file mmc1.pdf]

Biophysical Journal, Volume *123*

Supplemental information

Morphology and intervesicle distances in condensates of synaptic vesicles and synapsin

Charlotte Neuhaus, Jette Alfken, Jakob Frost, Lauren Matthews, Christian Hoffmann, Marcelo Ganzella, Dragomir Milovanovic, and Tim Salditt

Synaptic vesicles

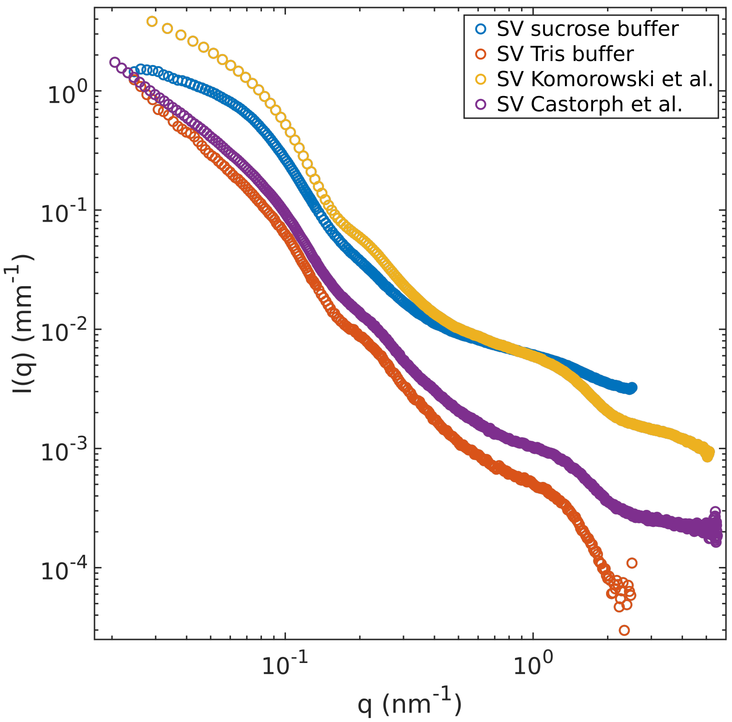

Figure S1: Comparison of different scattering curves obtained on synaptic vesicles in previous studies by Komorowski et al. (yellow) (1) and Castorph et al. (violet) (2) and in this study (blue,red). Especially in the intermediate q-range, all curves exhibit a similar functional form sharing characteristic modulations. The deviations in the small q-range are caused by the different amount of contamination by larger membranous particles.

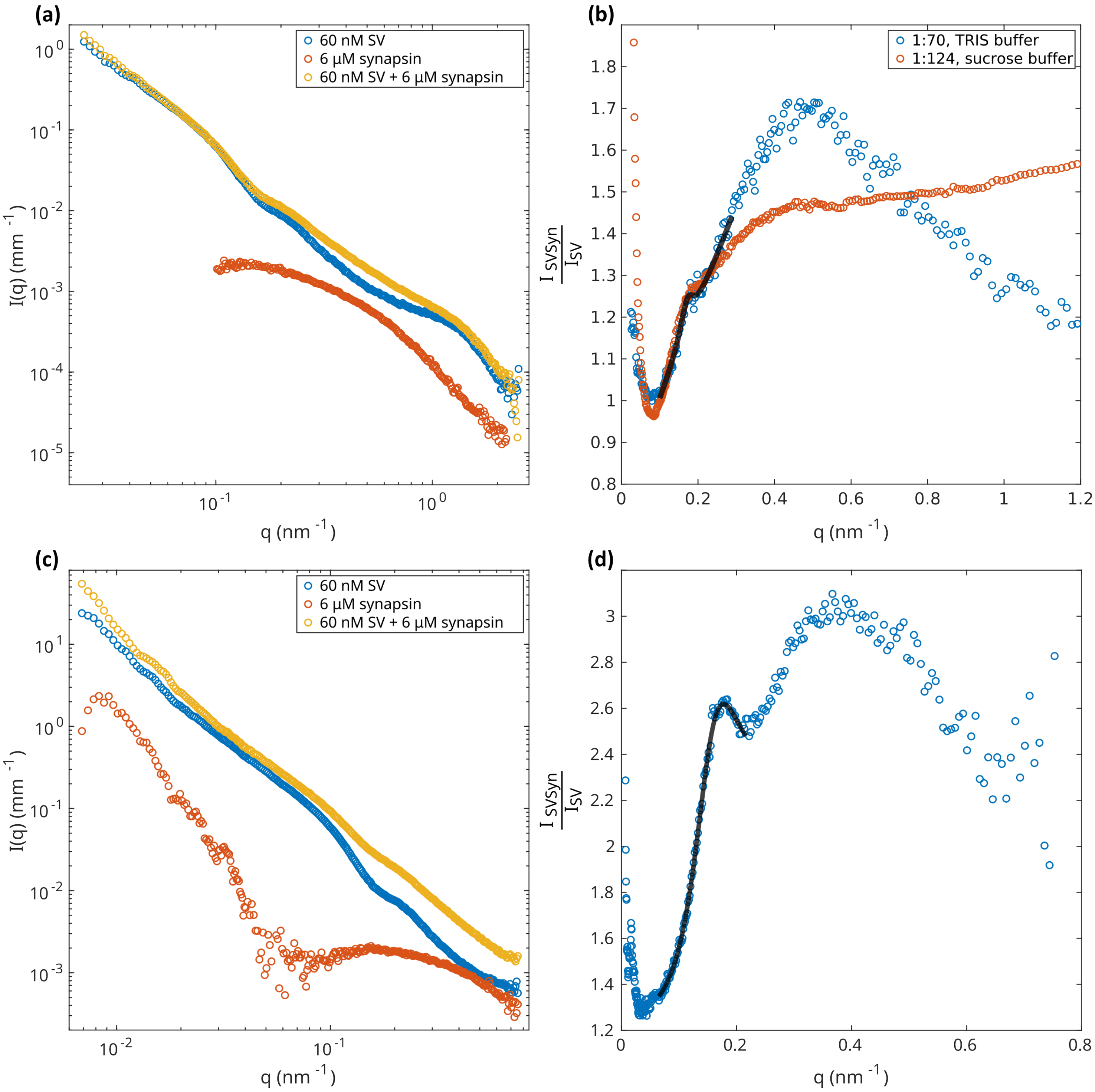

Figure S2: Scattered intensities and corresponding structure factors for measurements on SV-synapsin condensates in TRIS buffer. (a) Scattered intensities of samples containing 60 nm SVs in TRIS buffer and 6 μM synapsin, corresponding to a $P/L = 1 : 70$, measured at a detector distance of 3 m and an acquisition time of 0.1 s. (b) Structure factor a of the scattering curves shown in (a) (blue) and least-square fit with the distribution introduced in Eq. 3 compared to the structure factor of the sample with $P/L = 1 : 124$ previously shown in Fig. 4 (b)(red). For a better comparison of the structure factors, the 1:124 curve was vertically shifted. (c) Scattered intensity of a sample with $P/L = 1 : 70$ measured at a detector distance of 10 m with an acquisition time of 0.1 s. (d) Structure factor obtained from the scattering curves in (c) and least square fit with distribution introduced in Eq. 3.

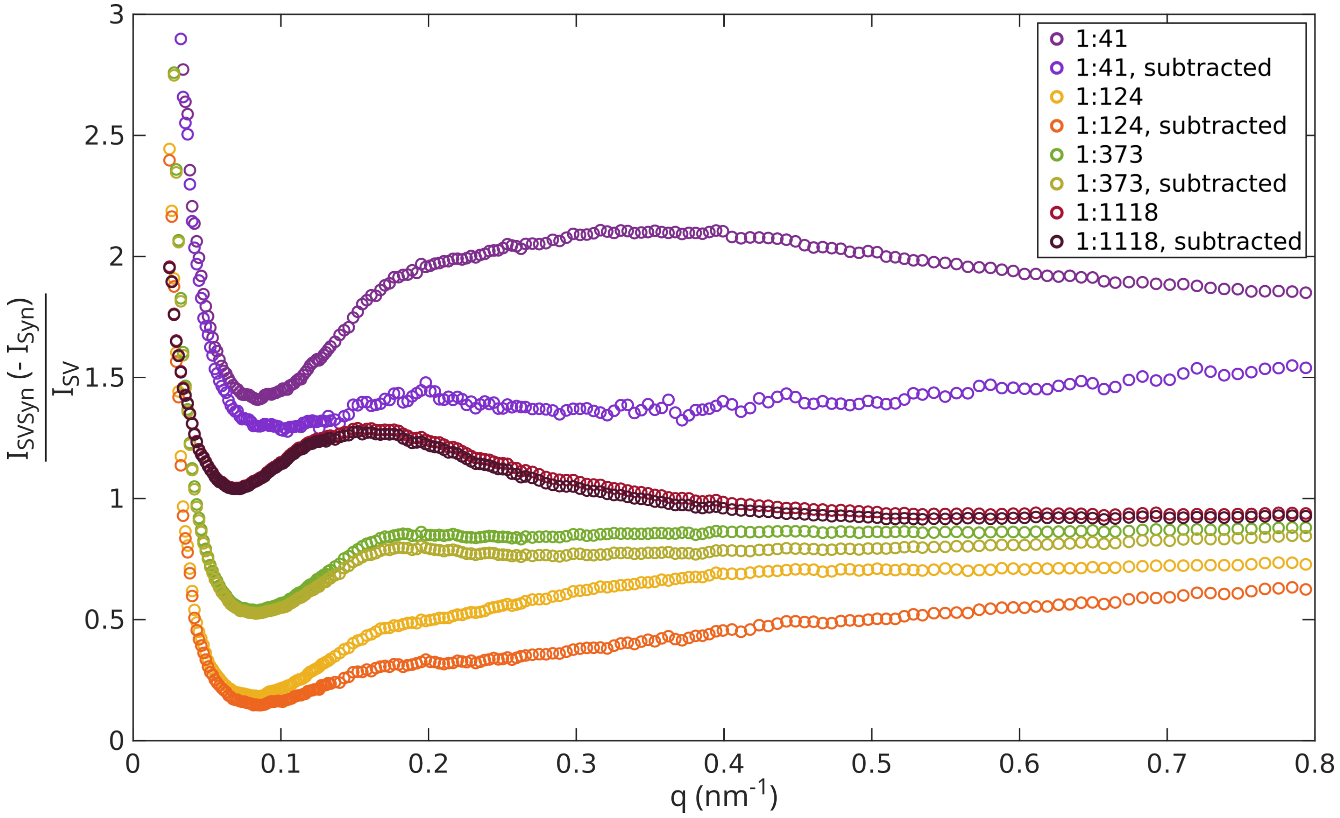

Figure S3: Comparison of structure factors condensates of SVs in sucrose and synapsin with and without the subtraction of synapsin, the curves are shifted vertically for clarity. The structure factors are calculated as described in the result section. The structure factor for a $P/L = 1 : 41$ significantly changes with the subtraction of synapsin and a peak that was previously not visible emerges. For the lower P/Ls the change becomes less significant and decreases with a decreasing P/L.

Error estimation

The fitting errors and covariance matrices for the nonlinear least-square fits shown in Fig. 3 were determined as described in (1). Onehundred pseudo-realizations of the 'experimental' data were generated from randomly drawn values from a normal distribution around each measured data point. The standard derivation of the distribution was described by the experimental errors. By fitting the synaptic vesicle model to the generated data, a parameter vector was obtained, which is then used to calculate the fit errors for each model parameter as well as the covariance matrix. The resulting model parameters and corresponding fit errors are tablated in Tab. S1, the correlation matrices for SVs in sucrose and SVs in TRIS buffer are tabulated in Tab. S2 and S3, respectively. An explanation of the parameters of the covariance matrix is given in Tab. S4.

Model fit parameter	SV sucrose buffer	SV TRIS buffer	Unit
$\rho_{\text{in}}, \rho_{\text{out}}$	35.5 ± 0.3	46.8	e^- nm^{-3}
ρ_{tail}	−40.14 ± 0.3	-28.8	e^- nm^{-3}
$t_{\text{in}}, t_{\text{out}}$	1.86 ± 0.02	1.69 ± 0.03	nm
t_{tail}	0.97 ± 0.02	1.07 ± 0.03	nm
R_g^{in}	2.28 ± 0.04	1.87 ± 0.1	nm
R_g^{out}	4.8 ± 0.05	5.25 ± 0.12	nm
$N_c^{\text{in}}/(4\pi(R-D-R_g^{\text{in}})^2)$	0.028 ± 0.0012	0.031 ± 0.003	nm^{-2}
$N_c^{\text{out}}/(4\pi(R+R_g^{\text{in}})^2)$	0.00096 ± 0.0001	0.0008 ± 0.0001	nm^{-2}
ρ_c	40.7 ± 0.03	52.1	e^- nm^{-3}
R	16.95	16.95	nm
σ_{R}	3.92	3.92	nm
Amplitude	242.7 ± 3.2	232.6 ± 1.6	Arb. units
R_{large}	179.4 ± 6.6	261.6 ± 6.2	nm
$\sigma_{\text{R,large}}$	46.2 ± 2.3	78.6 ± 2.3	nm
$\text{Amplitude}_{\text{large}}$	1.91 ± 0.11	1.25 ± 0.05	Arb. units
Scale	0.79 ± 0.02	0.091 ± 0.004	-
Constant background	0.0028 ± 0.0001	$-3.6 \cdot 10^{-6} \pm 6 \cdot 10^{-6}$	1/mm

Table S1: Model parameters and corresponding fit errors resulting from the least-squares fit of the N=100 generated pseudo experimental data using the synaptic vesicle model. The fitting was performed as described in the results section. The maximum number of iterations was set to 500.

	P_1	P_2	P_3	P_4	P_5	P_6	P_7	P_8	P_9	P_{10}	P_{11}	P_{12}	P_{13}
P_1	1	-0.91	0.96	-0.66	-0.57	0.76	-0.06	0.75	0.53	-0.22	0.17	0.21	0.16
P_2	-0.91	1	-0.98	0.8	0.36	-0.55	0.06	-0.86	-0.63	0.27	-0.2	-0.28	-0.29
P_3	0.96	-0.98	1	-0.74	-0.45	0.66	-0.05	0.83	0.64	-0.27	0.21	0.27	0.29
P_4	-0.66	0.8	-0.74	1	0.25	-0.25	0.03	-0.98	-0.55	0.12	-0.08	-0.15	-0.23
P_5	-0.57	0.36	-0.45	0.25	1	-0.7	-0.49	-0.34	0.09	-0.15	0.04	0.18	0.36
P_6	0.76	-0.55	0.66	-0.25	-0.7	1	-0.09	0.38	0.11	-0.17	0.14	0.13	-0.21
P_7	-0.06	0.06	-0.05	0.03	-0.49	-0.09	1	-0.01	0.07	0.27	-0.08	-0.29	0.13
P_8	0.75	-0.86	0.83	-0.98	-0.34	0.38	-0.01	1	0.59	-0.14	0.1	0.15	0.25
P_9	0.53	-0.63	0.64	-0.55	0.09	0.11	0.07	0.59	1	-0.31	0.3	0.31	0.91
P_{10}	-0.22	0.27	-0.27	0.12	-0.15	-0.17	0.27	-0.14	-0.31	1	-0.97	-0.95	-0.26
P_{11}	0.17	-0.2	0.21	-0.08	0.04	0.14	-0.08	0.1	0.3	-0.97	1	0.91	0.26
P_{12}	0.21	-0.28	0.27	-0.15	0.18	0.13	-0.29	0.15	0.31	-0.95	0.91	1	0.25
P_{13}	0.16	-0.29	0.29	-0.23	0.36	-0.21	0.13	0.25	0.91	-0.26	0.26	0.25	1

Table S2: Correlation matrix for the varied fit parameters obtained for SVs in sucrose buffer.

	P_1	P_2	P_3	P_4	P_5	P_6	P_7	P_8	P_9	P_{10}	P_{11}	P_{12}
P_1	1	-0.87	0.96	-0.7	-0.93	0.85	-0.61	0.8	0.96	-0.77	0.76	0.73
P_2	-0.87	1	-0.95	0.8	0.7	-0.67	0.44	-0.88	-0.82	0.76	-0.65	-0.72
P_3	0.96	-0.95	1	-0.77	-0.86	0.81	-0.56	0.89	0.94	-0.8	0.74	0.75
P_4	-0.7	0.8	-0.77	1	0.53	-0.47	0.48	-0.96	-0.75	0.72	-0.67	-0.68
P_5	-0.93	0.7	-0.86	0.53	1	-0.9	0.53	-0.66	-0.93	0.68	-0.74	-0.65
P_6	0.85	-0.67	0.81	-0.47	-0.9	1	-0.56	0.61	0.9	-0.61	0.62	0.61
P_7	-0.61	0.44	-0.56	0.48	0.53	-0.56	1	-0.51	-0.63	0.58	-0.53	-0.61
P_8	0.8	-0.88	0.89	-0.96	-0.66	0.61	-0.51	1	0.83	-0.77	0.7	0.71
P_9	0.96	-0.82	0.94	-0.75	-0.93	0.9	-0.63	0.83	1	-0.78	0.77	0.75
P_{10}	-0.77	0.76	-0.8	0.72	0.68	-0.61	0.58	-0.77	-0.78	1	-0.94	-0.89
P_{11}	0.76	-0.65	0.74	-0.67	-0.74	0.62	-0.53	0.7	0.77	-0.94	1	0.82
P_{12}	0.73	-0.72	0.75	-0.68	-0.65	0.61	-0.61	0.71	0.75	-0.89	0.82	1

Table S3: Correlation matrix for the varied fit parameters obtained for SVs in TRIS buffer.

P_1	$D/2$, half thickness of the shell
P_2	$N_c^{\text{out}}/(4\pi(R+R_g^{\text{in}})^2)$
P_3	R_g^{out}
P_4	$N_c^{\text{in}}/(4\pi(R-D-R_g^{\text{in}})^2)$
P_5	Scale
P_6	Constant background
P_7	Amplitude
P_8	R_g^{out}
P_9	Fraction $t_{\text{headgroup}}/D$
P_{10}	R_{large}
P_{11}	$\text{Amplitude}_{\text{large}}$
P_{12}	R_{large}
P_{13}	excess scattering of sucrose buffer (compared to water)

Table S4: Explanation of the fit parameters P_i in the correlation matrix.

Lipid Vesicles

Additional to SVs, condensates of lipid vesicles and synapsin were measured. Figure S4 (a) shows the scattered intensities of samples containing 6 μM synapsin, 1.56 mM LV4 and condensates thereof with $P/L = 1 : 260$ measured at a sample distance of 3 m. For the pure LV4 sample, a curve measured at a concentration of 5 mM LV4 was used and the intensity was scaled to fit the intensity of a 1.56 mM LV4 sample.

To obtain more quantitative information on the vesicles, a model for the scattered intensity is fitted to the data. For this fit, the model described in (3, 4) was used. In the model, the vesicles are described as polydisperse spherical particles with a Gaussian distributed polydispersity with mean radius R and width σ_R. The radius is described as the radius to the center of the lipid bilayer. The radial electron density profile of the lipid bilayer is described by three Gaussians as

$$\rho(r) = \sum_{i=1}^{3} \rho_i \exp\left(\frac{-(z - z_i)^2}{2t_i^2}\right), \tag{1}$$

representing the head- and tail-regions of the lipids, with relative electron density ρ_i, position z_i and width t_i. The profile is assumed to be symmetric, so $\rho_\mathrm{h} = \rho_\mathrm{h,in} = \rho_\mathrm{h,out}$, $\sigma_\mathrm{h} = \sigma_\mathrm{h,in} = \sigma_\mathrm{h,out}$ and $z_\mathrm{h} = z_\mathrm{h,in} = -z_\mathrm{h,out}$. The position of the tail-region is fixed at $z_\mathrm{t} = 0$ and the relative electron density at $\rho_\mathrm{t} = -1$, all other model parameters were freely varied. A powerlaw-corrected background described by $I_\mathrm{background}(q) = c_2\, q^{-c_3} + c_4$ was added to the model intensity. The last 330 points were not included in the fitting process. To fit the model to the data a least squares fit with the a reduced χ^2 cost function (see Eq. 2) was performed. For fitting, the lsqnonlin-function of the MATLAB R2020a Optimization toolbox was used, for numerical implementation of the model was performed as described in (5). The resulting model fit curve and the resulting electron density profile are shown in Fig. S4 (a), the corresponding model parameters are tabulated in Tab S5.

The structure factor calculated from the scattering curves in (a) are shown in (b) and (c). While in (c) the intensity of synapsin is subtracted form the intensity of the cluster before the division, no subtraction was performed in (b). Both structure factors show a peak at $q \approx 0.3\,\mathrm{nm}^{-1}$, to determine the exact peak position, a skewed Cauchy-Lorentz distribution (see Eq. 3) was fitted to the data. The resulting model parameters are shown in Tab. S6. Figure S4 (d) shows the scattering curves for samples containing 6 μM synapsin, 11 mM LV4 and condensates thereof measured at a sample distance of 10 m. The structure factors without and with subtraction of synapsin are shown in (e) and (f) respectively. Again, a skewed Cauchy-Lorentz distribution was fitted to the structure factors, the resulting model parameters are shown in Tab. S6.

Model fit parameter	value
t_h	0.34
t_t	0.74
ρ_h	1.42
ρ_t	1 (fixed)
z_h	±1.99
z_t	0 (fixed)
R (nm)	13.85
σ_R	8.1
scale	$1.3 \cdot 10^{-6}$
c_2	$7.4 \cdot 10^{-9}$
c_3	5.2
c_4	$1.6 \cdot 10^{-4}$

Table S5: Model parameters for the least-squares fit of the scattered intensity of 5 mM LV4, shown in Fig. S4 (a) using the model described in (4). The vesicles are described as Gaussian distributed polydisperse particles with a mean radius R and a width σ_R. The electron density profile is described by three Gaussians with amplitude (relative electron densities) ρ_i, width t_i and position $z_i, i \in \{h_{in}, h_{out}, t\}$. The profile is assumed to be symmetric. Additional background was described by a powerlaw correction $I_{background} = c_2 q^{-c_3} + c_4$.

Model fit parameter	3 m (Fig. S4 (b)) no subtraction	3 m, (fig. S4 (c)) synapsin subtracted	10 m (fig. S4 (e)) no subtraction	10 m (fig. S4 (f)) synapsin subtracted
σ	0.119 ± 0.003	0.121 ± 0.004	0.16 ± 0.06	0.20 ± 0.02
μ	0.313 ± 0.003	0.315 ± 0.003	0.299 ± 0.003	0.142 ± 0.007
λ	0.8 ± 0.11	0.66 ± 0.16	0.78 ± 0.092	0.3 ± 0.5
scale	10.82 ± 0.18	10.96 ± 0.22	2.26 ± 0.08	0.73 ± 0.05

Table S6: Parameters resulting from the least squares fits to the calculated LV4-synapsin structure factors, shown in Fig. S4 using a skewed Cauchy-Lorentz distribution (Eq. 3). σ describes the HWHM of the distribution, μ the position of the peak and λ the skewness of the distribution.

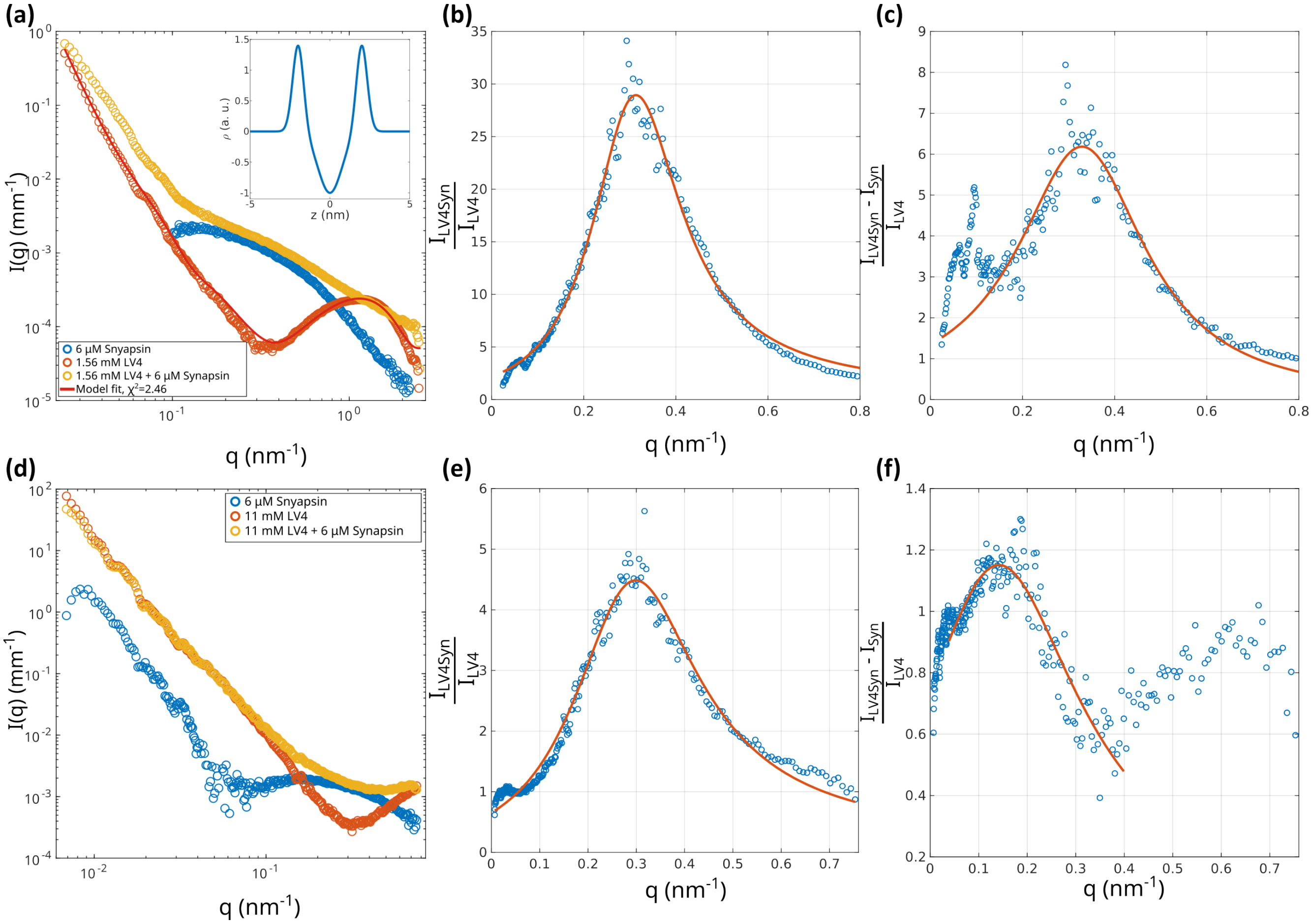

Figure S4: Scattered intensities and corresponding structure factor curves for measurements on LV4-synapsin condensates. (a) Scattering curves of LV4-synapsin condensates at concentrations of 6 μM synapsin and 1.56 mM LV4 (P/L=1:260) measured at a detector distance of 3 m. The acquisition time of the pure synapsin sample was 0.1 s, of the pure LV4 sample 0.5 s and of the condensate sample 0.2 s. The pure LV4 sample was measured at a concentration of 5 mM and the intensity was scaled to fit the intensity of a 1.56 mM LV4 sample. (b) Structure factor of the LV4-synapsin condensates calculated from the scattering curves in (a). The intensity of pure synapsin is not subtracted form the intensity of the condensates. (c) Structure factor of the LV4-synapsin condensates calculated from the scattering curves in (a). In contrast to (b), the intensity of pure synapsin is subtracted before division by the intensity of SVs. (e) Scattering curves of LV4-synapsin condensates at concentrations of 6 μM synapsin and 11 mM LV4 (P/L=1:1833) at a detector distance of 10 m. Even though there is almost no indication of vesicle clustering in the low q-range, which is attributed to the excess of free lipid vesicles at such high lipid concentrations, the signal of the fraction of condensates is visible in the high q-range. This is also visible in the resulting structure factors, which are shown without (e) and with (f) the subtraction of synapsin. Even though an excess of synapsin is not reasonable for such high lipid concentrations, we show this curve for completeness. All samples were measured at an acquisition time of 0.1 s. (e) Structure factor of the LV4-synapsin condensates calculated form the scattering curves in (d). (f) Structure factor obtained from the curves in (d), in contrast to (e), the pure synapsin intensity is subtracted before division. A skewed Cauchy-Lorentz distribution (see Eq. 3) was fitted to all structure factor curves. The model parameters for these fits are shown in Tab. S6.

SUPPLEMENTAL REFERENCES

1. Komorowski, K., J. Preobraschenski, M. Ganzella, J. Alfken, C. Neuhaus, R. Jahn, and T. Salditt, 2022. Neurotransmitter uptake of synaptic vesicles studied by X-ray diffraction. *European Biophysics Journal* 51:465–482. https://doi.org/10.1007/s00249-022-01609-w.

2. Castorph, S., D. Riedel, L. Arleth, M. Sztucki, R. Jahn, M. Holt, and T. Salditt, 2010. Structure Parameters of Synaptic Vesicles Quantified by Small-Angle X-Ray Scattering. *Biophysical Journal* 98:1200–1208. https://www.sciencedirect.com/science/article/pii/S0006349509060913.

3. Pabst, G., M. Rappolt, H. Amenitsch, and P. Laggner, 2000. Structural information from multilamellar liposomes at full hydration: full q-range fitting with high quality x-ray data. *Phys Rev E* 62:4000–4009.

4. Brzustowicz, M. R., and A. T. Brunger, 2005. X-ray scattering from unilamellar lipid vesicles. *Journal of Applied Crystallography* 38:126–131. https://doi.org/10.1107/S0021889804029206.

5. Komorowski, K., A. Salditt, Y. Xu, H. Yavuz, M. Brennich, R. Jahn, and T. Salditt, 2018. Vesicle Adhesion and Fusion Studied by Small-Angle X-Ray Scattering. *Biophysical Journal* 114:1908–1920. https://www.sciencedirect.com/science/article/pii/S0006349518303412.
