# Supplementary material for: Morphology and intervesicle distances in condensates of synaptic vesicles and synapsin
Source: Biophys J. 2024 Nov 8;123(23):4123–34. doi: 10.1016/j.bpj.2024.11.004 (PMC11628805; doi:10.1016/j.bpj.2024.11.004)
Supplement: Document S2. Article plus supporting material [file mmc2.pdf]

Biophysical Journal
Article

Biophysical Society

Morphology and intervesicle distances in condensates of synaptic vesicles and synapsin

Charlotte Neuhaus,[1] Jette Alfken,[1] Jakob Frost,[1] Lauren Matthews,[2] Christian Hoffmann,[3] Marcelo Ganzella,[4] Dragomir Milovanovic,[3] and Tim Salditt[1,*]
[1]Institut für Röntgenphysik, Göttingen, Germany; [2]The European Synchrotron Radiation Facility, Grenoble, France; [3]Laboratory of Molecular Neuroscience, German Center for Neurodegenerative Diseases (DZNE), Berlin, Germany; and [4]Laboratory of Neurobiology, Max Planck Institute for Multidisciplinary Sciences, Göttingen, Germany

ABSTRACT Synaptic vesicle clusters or pools are functionally important constituents of chemical synapses. In the so-called reserve and the active pools, neurotransmitter-loaded synaptic vesicles (SVs) are stored and conditioned for fusion with the synaptic membrane and subsequent neurotransmitter release during synaptic activity. Vesicle clusters can be considered as so-called membraneless compartments, which form by liquid-liquid phase separation. Synapsin as one of the most abundant synaptic proteins has been identified as a major driver of pool formation. It has been shown to induce liquid-liquid phase separation and form condensates on its own in solution, but also has been shown to integrate vesicles into condensates in vitro. In this process, the intrinsically disordered region of synapsin is believed to play a critical role. Here, we first investigate the solution structure of synapsin and SVs separately by small-angle x-ray scattering. In the limit of low momentum transfer q, the scattering curve for synapsin gives clear indication for supramolecular aggregation (condensation). We then study mixtures of SVs and synapsin-forming condensates, aiming at the morphology and intervesicle distances, i.e., the structure of the condensates in solution. To obtain the structure factor $S(q)$ quantifying intervesicle correlation, we divide the scattering curve of condensates by that of pure SV suspensions. Analysis of $S(q)$ in combination with numerical simulations of cluster aggregation indicates a noncompact fractal-like vesicular fluid with rather short intervesicle distances at the contact sites.

SIGNIFICANCE Synapsin as one of the most abundant synaptic proteins is the main protein believed to induce and maintain synaptic pools. Here, we show that synapsin and synaptic vesicles condense in vitro to a complex fluid with close contacts between vesicles but an overall noncompact morphology, which may facilitate diffusion and transport of metabolites.

INTRODUCTION

Communication between synapses relies on synaptic vesicles (SVs) as highly specialized trafficking organelles (1). These small neurotransmitter-filled vesicles with a radius $R \approx 20$ nm are enclosed by a lipid bilayer packed with a plethora of proteins underlying its transport, signaling, and release functions (2). In a synapse, SVs are organized in distinct vesicle pools (1,3). The main protein associated with pool formation, i.e., the clustering of vesicles, is believed to be the neuron-specific phosphoprotein synapsin I (1,4). In this study, we focus on the synapsin Ia isoform, simply referred to as synapsin below. With approximately 6606 copies per synaptic bouton, synapsin is one of the most abundant proteins in synapses and can be found in all presynaptic terminals (5,6). Its N-terminal (domains A–C, residues 1–420) can penetrate into the hydrophobic core of a phospholipid bilayer and bind to phospholipid membranes of SVs (7). The C-terminal (domains D–E, residues 420–705) consists of large intrinsically disordered regions, i.e., regions lacking a fixed secondary structure, and associates with protein components of SVs (7,8). Synapsin has also been shown to undergo liquid-liquid phase separation in vitro, forming distinct condensates in aqueous environments (9). It was also shown that these condensates can recruit small charged lipid vesicles (LVs) (9) as well as SVs (10). In fact, the presence of SVs even accelerates the formation of these condensates (10). The mesoscale organization of SV-synapsin condensates is influenced by the protein/lipid ratio (P/L) and also

Submitted July 26, 2024, and accepted for publication November 4, 2024.
*Correspondence: tsaldit@gwdg.de
Editor: Frederick Heberle.
https://doi.org/10.1016/j.bpj.2024.11.004
© 2024 The Authors. Published by Elsevier Inc. on behalf of Biophysical Society.
This is an open access article under the CC BY license (http://creativecommons.org/licenses/by/4.0/).

the presence of other synaptic proteins such as α-synuclein (10–12). Recently, we showed that the morphology of the condensates, as observed by fluorescence light microscopy changes from spherical condensates to more fractally appearing shapes with decreasing P/L (11). While the macrostructure of these condensates can be readily assessed, for example, by fluorescence light microscopy, information about the microstructure, in particular concerning the structural organization of vesicles within the condensates, is much more challenging to obtain. Cryogenic electron microscopy (cryo-EM) has revealed the formation of pronounced adhesion zones with flattened bilayer contact areas in condensates of LVs and synapsin, while no such adhesion zones were observed in SV-synapsin condensates (11). However, the sample volume that can be probed by cryo-EM is often limited, and from isolated images it can be difficult to infer statistical information. Regarding the morphology of lipid samples, it is also critical to avoid partial dehydration in the plunge freezing process, notably in the chamber where excess liquid is blotted before cryofixation. Furthermore, in cryofixated samples, active states are not accessible, and buffer conditions cannot be changed. Solution small-angle x-ray scattering (SAXS), on the other hand, offers the required resolution and is compatible with physiological conditions. While the rather indirect nature of the measurements poses its own challenges in particular regarding modeling and analysis, the inherent averaging of SAXS as an ensemble technique can be as much a limitation as an advantage. For this reason, cryo-EM and SAXS are highly complementary for studies of lipid assemblies in particular. Previously, SAXS has been used by our group to investigate the size, polydispersity, and structure of purified SVs. These studies yielded information on the size and electron density of the protein shells and the lipid bilayer, as well as the shape transformation occurring during uptake of neurotransmitters (13,14). SAXS was also previously used to study the morphological changes in protein solutions during liquid-liquid phase separation (15).

In this work, we have performed SAXS experiments on the minimal in vitro model of condensates consisting of synapsin and SVs, as schematically shown in Fig. 1. Our study aims at the morphology of these condensates at a resolution resolving intervesicle distances, and as a function of P/L values. We mainly focus on condensates of SVs, but also show results of LV condensates. The goal

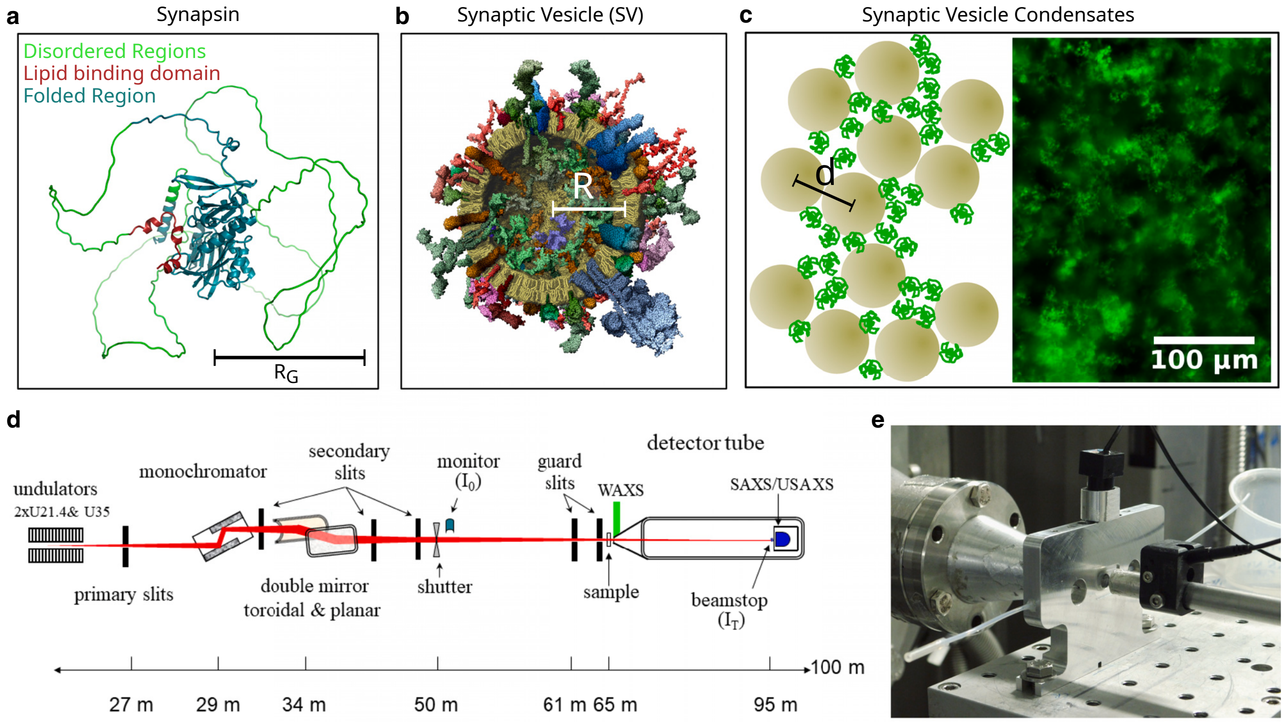

FIGURE 1 (*a*) Structure of synapsin as predicted by AlphaFold (16). The disordered regions and lipid binding domain are indicated in green and red, respectively. (*b*) Schematic structure of SV as shown in (2). The vesicle radius R and the density profile including the bilayer and the protein shells can be determined from the SV form factor measured by solution scattering. (*c*, *left*) Schematic structure of a synapsin vesicle condensate, with the average distance d between neighboring vesicles, which can be inferred from the structure factor. (c, *right*) Fluorescence microscopy image (20×) of condensates formed from 6 μM EGFP-labeled synapsin and 15.6 mM lipid vesicles ($P/L = 1:2600$), adapted from (11). While a fractal appearance is readily visible on large scales, the present work addresses the condensate morphology on sub-μm scales by way of SAXS. (*d*) Schematic layout and optical components of the ID02 beamline at ESRF as shown in (17). The SAXS data was recorded at two different sample-detector distances of $z = 3$ m and $z = 10$ m, respectively. (*e*) Photograph of the beamline near the sample, showing the sample chamber including the flowthrough capillary.

is to determine the structural organization of the vesicles inside the condensates in terms of intervesicle distances and packing. This is achieved by extracting and modeling the structure factor $S(q)$ from the measured intensity $I(q) \propto S(q)F(q)$ as a function of momentum transfer q, for known (measured) single-particle form factor $F(q) = |f(q)|^2$.

The article is organized as follows: after this introduction, the materials and methods describes the sample preparation and the SAXS measurements. The results first focuses on the measurement of pure synapsin as well as SVs, before presenting the findings on SV-synapsin condensates. After this, a model for the inter condensate vesicle distribution is introduced, to adequately interpret the observed structure factor. The article then closes with a brief conclusion and outlook section.

MATERIALS AND METHODS

Sample preparation

Synapsin I

EGFP-labeled Synapsin I was expressed in and purified from Expi293 cells as described in (9,10). Purified synapsin was solved in a buffer solution consisting of 25 mM Tris-HCl, 150 mM NaCl, and 0.5 mM TCEP (pH 7.4) at 4°C, herein referred to as TRIS buffer. After purification, synapsin was snap-frozen in liquid nitrogen and was kept frozen at -80°C or on liquid N_2. For the measurements, synapsin was thawed on ice.

Liposomes

For liposome formation DOPC (1,2-dioleoyl-*sn*-glycero-3-phosphocholine), DOPS (1,2-dioleoyl-*sn*-glycero-3-phospho-L-serine), DOPE (1,2-dioleoyl-*sn*-glycero-3-phosphoethanolamine), and cholesterol were purchased from Avanti Polar Lipids (Alabaster, AL) in powder form. Lipids were then dissolved in chloroform and mixed in the desired concentrations. In this study, a mix of 55 mol % DOPC, 20 mol % DOPS, 15 mol % DOPE, and 10 mol % cholesterol was used as a coarse model for the charge distribution and lipid composition of SVs, following the protocol of (9). Chloroform was evaporated using a stream of N_2 and a vacuum oven, and the resulting lipid film was rehydrated in TRIS buffer. Vesicles were then formed by 10 freeze (liquid N_2) and thaw (37° C water bath) cycles. This was followed by 21 extrusion cycles through a polycarbonate pore membrane with pore size of 50 nm using the Avanti Polar Lipids Mini Extruder (Alabaster). These four-component LVs will be denoted as LV4 in the following.

SVs

SVs were purified from rat brain as described in (2), and vesicles were resuspended in a buffer solution. After purification, SVs were snap-frozen and kept frozen at -80°C or on liquid N_2. For the measurements, SVs were thawed on ice. In this study, two different preparations of SVs were used. The first preparation resulted in an SV concentration of $c_{\mathrm{SV,sucrose}} \approx$ 240 nM and vesicles were dissolved in a sucrose buffer (320 mM sucrose, 10 mM HEPES [pH 7.4]), the second preparation resulted in an SV concentration of $c_{\mathrm{SV,Tris}} \approx 320$ nM and the vesicles were stored in the TRIS buffer also used for synapsin.

Vesicle condensates

For condensate formation, synapsin and (synaptic) vesicles were mixed on ice in the desired concentrations and allowed to incubate for a few minutes.

SAXS

SAXS experiments were carried out at the ID02 beamline at European Synchrotron Radiation Facility (ESRF) in Grenoble, France (17,18), at settings tabulated in Table 1. The beam was monochromized using a Si(111) crystal to a photon energy of 12.233 keV. The cross section of the collimated undulator beam was set to 500 μm by secondary slits. The photon flux near the sample was $3.31 \times 10^{12}\ \frac{\mathrm{ph}}{\mathrm{s}}$. The sample was placed in the beam in a 1 mm biocompatible polycarbonate flowthrough capillary. Around 10 μL of sample solution was injected into the capillary. Between different samples, the capillary was thoroughly cleaned using Hellmanex (Hellma, Müllheim, Germany) and deionized water and subsequently dried using compressed air. On each sample, 5 different positions with a lateral distance of at least 0.5 mm were measured. On each position, 10 shots with an acquisition time of 0.1/0.5 s were taken. The scattered signal was measured using the single-photon counting detector Eiger2 4M (Dectris, Baden, Switzerland) 3/10 m behind the sample. During the measurements, the 2D diffraction patterns were simultaneously normalized and azimuthally averaged by the online data reduction tool implemented at the beamline (18). Further data reduction was performed using the SAXS-utilities2 toolbox (19). The following workflow was adopted: first, curves measured at the same position of the capillary were averaged. Second, the average background taken at the same position on the capillary was subtracted. Third, the data were dynamically rebinned using the tool implemented in SAXS-utilities. Finally, the averaged scattering curve over all positions was calculated and used for further analysis. During the processing, it was assured that data measured on different positions had the same shape and no radiation damage was visible. Samples that were measured at both sample-to-detector distances were merged together using the merging tool implemented in SAXS-utilities. For this, the data were plotted and the region in which the curves show similar behavior was selected as the merge region. The intensity of the brighter curve was chosen as the new intensity. The beamtime data (SC 5112) is publiclly availiable under https://doi.org/10.15151/ESRF-ES-450256620 .

RESULTS AND DISCUSSION

We first present and discuss the SAXS results of the constituent solutions, i.e., pure synapsin and pure SVs, before we address the condensates formed by mixing the two solutions. Finally, we discuss the structure factor of the condensates in the light of fractal cluster simulations.

Synapsin

Fig. 2 shows different representations of the signal measured on a sample of 6 μM synapsin in TRIS buffer. In Fig. 2 *a* the azimuthally averaged and background subtracted intensity I is shown as a function of the scattering

TABLE 1 Beamline settings and parameters

Parameter	Value
Photon energy E_{ph} (keV)	12.233
Photon flux ($\frac{\mathrm{ph}}{\mathrm{s}}$)	3.31×10^{12}
Monochromator	Si(111)
Beam size (mm^2)	0.5×0.5
Sample detector distance (m)	3/10
Acquisition time (s)	0.1/0.5
Detector	Eiger2 4M
Pixel size (μm^2)	75×75

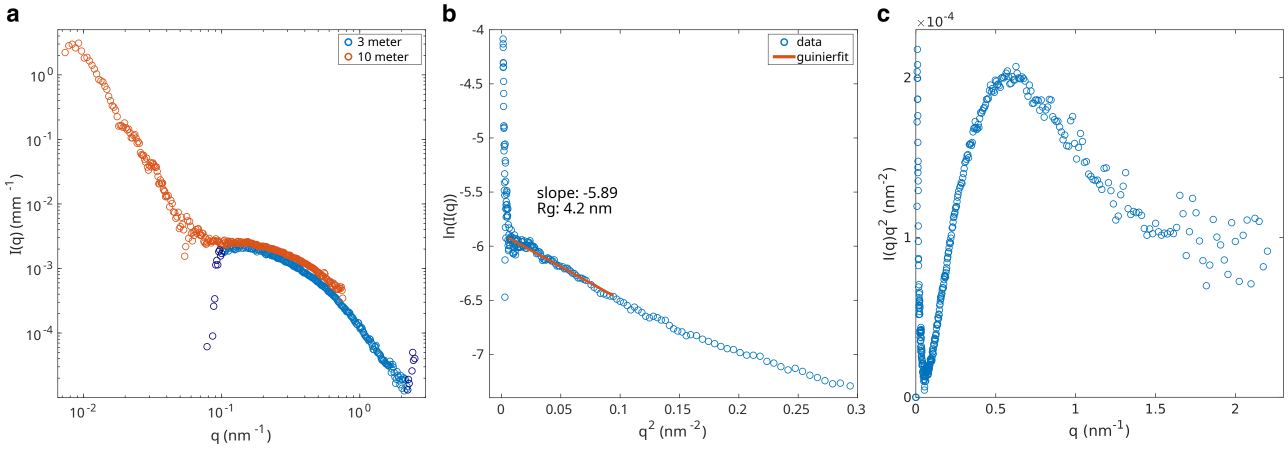

FIGURE 2 Solution scattering of synapsin (6 μM, TRIS buffer), shown in different representations. (*a*) Azimuthal average of the data measured at a sample-detector distance of 3 and 10 m with an acquisition time of 0.1 s after background subtraction. The darker colored points in the 3 m curve were discarded due to noise. For further analysis, a merged curve with contributions from both distances was used. The low q data are indicative for the formation of condensates. (*b*) Guinier representation of the merged curve, the radius of gyration is determined to $R_g = 4.2$ nm. The fitting was performed in a range between $q_{min} = 0.075$ nm^{-1} and $q_{max} = 0.31$ nm^{-1}. (*c*) Kratky representation of the merged curve. The shape of the curve—which shows a distinct maximum that decays into a plateau-like region instead of a bell-shaped curve—indicates a solution of partially unfolded proteins.

vector $q = 4\pi/\lambda \sin(\theta)$ for both sample-to-detector distances. The darker colored points of the 3 m curve were discarded from further analysis, because the signal is influenced by excessive background subtraction and instrumental noise in this region. In the region, where the two curves overlap, they exhibit a similar behavior. For further analysis, the two curves are merged. The increase in intensity in the lower q region ($q < 0.08$ nm^{-1}) indicates a condensation of synapsin. This is consistent with previous studies using fluorescence microscopy, in which condensates of synapsin could be observed at these protein concentrations (11). Despite the fact that the condensation presumably induced loose gel-like interactions, we can identify a Guinier regime, dominated by the molecular size, or more precisely the radius of gyration R_g of synapsin. In Fig. 2 *b*, the Guinier representation of the merged intensity curve is shown. Using the standard relation for the intensity decay in this region

$$I(q) = I_0 e^{-q^2 R_g^2/3} \tag{1}$$

we can determine the radius of gyration R_g. Here, I_0 denotes the forward scattering intensity in the plateau region before the upturn at smaller q due to the condensation. For least-squares fitting, the q range 0.075 nm$^{-1} \leq q \leq 0.31$ nm^{-1} was selected, yielding $R_g = 4.2$ nm. This is close to the radius of gyration of 4.5 nm calculated using the rgyrate function (20) in PyMOL (21) and the predicted synapsin structure (16,22). In Fig. 2 *c*, the Kratky representation of the measured intensity is shown. The shape of the curve indicates that the sample is composed of partially unfolded proteins. This is in agreement with the fact that the synapsin C-terminal contains intrinsically disordered regions and therefore lacks a fixed secondary structure, i.e., is unfolded in this region.

SVs

Next we turn to measurements on dilute SV solutions. The scattered intensity of the different SV preparations, measured at a concentration of 240 nM for the SVs in sucrose buffer and at 60 nM for the SVs in TRIS buffer is shown in Fig. 3 *a*. A comparison with SV SAXS curves of previous studies by Komorowski et al. (14) and Castorph et al. (13) is shown in Fig. S1 in the supporting material. All scattering curves exhibit a similar functional form sharing the characteristic modulations. This can be taken as an indication that the preparations are of similar quality as in previous studies. The background of the SVs in sucrose buffer was not properly determined, so instead of the buffer background, the background of pure water was subtracted from the measured signal. For a quantitative analysis, the anisotropic SAXS model described in (13,14) was fitted to the measured intensity curves. The model describes the vesicles as polydisperse spherical particles with a bimodal size distribution of two Gaussians, accounting for the size distribution of the actual SVs, described by radius R, width σ_R and amplitude A, as well as a distribution for contamination by larger membranous particles, described by radius R_{large}, width σ_{large}, and amplitude A_{large}. Please note, that R_{large} and σ_{large} describe the contaminations only in an effective sense. Given the broad size range and the nonuniform spherical shape of the contaminant particles, an exact description of the size and shape of these particles is not possible. The radius of the vesicles is defined as the radius to the center of the bilayer. The radial electron density profile $\rho(r)$ of the

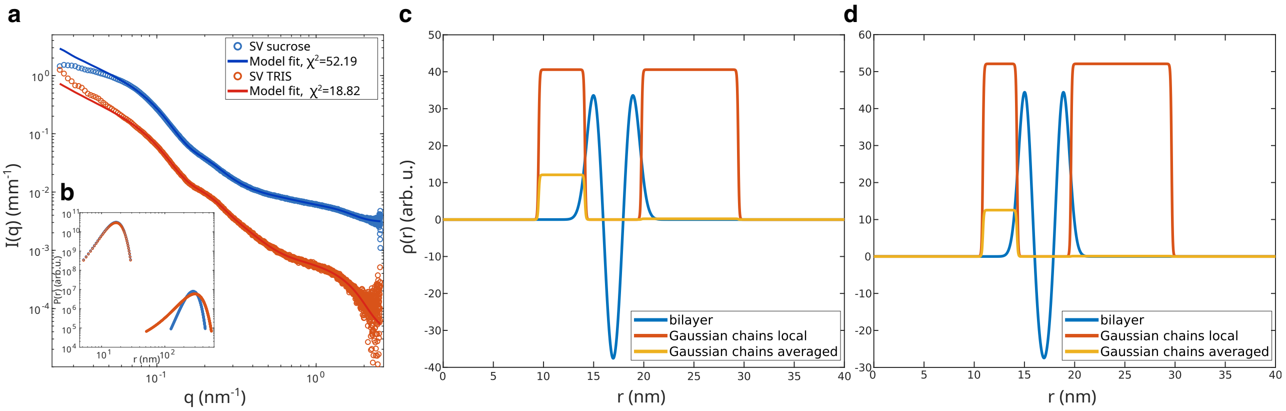

FIGURE 3 (*a*) Azimuthally averaged scattering intensity of the different SV preparations at a detector distance of 3 m and least-square fits. SVs in sucrose buffer were measured at a vesicle concentration of 240 nM, SVs in TRIS buffer were measured at a concentration of 60 nM. The first 30 points of the measured intensity were not included in the fitting process. For the SVs in sucrose buffer, the SAXS curve measured for pure water capillary was subtracted as a background. (*b*) Bimodal Gaussian size distribution obtained from the fits in (*a*). The bimodal distribution accounts for the size of the actual SVs (*smaller radii*) as well as contamination by larger membranous particles (*larger radii*). The size of the actual vesicles was kept constant during the fitting process, while the size of the larger particles was freely variable. (*c*) Excess electron density profile of the bilayer described by three Gaussians as well as the proteins described by Gaussian chains for SVs in sucrose buffer obtained from the fits in (*a*). The excess electron density of the proteins can be described by the typical local excess electron density of the protein patches (Gaussian chains local) or as the spherically averaged contribution (Gaussian chains averaged), which correspond to the excess electron density if the proteins were described by a spherical shell. (*d*) Excess electron density profiles for SVs in TRIS buffer.

vesicles bilayer is described by three Gaussians, with amplitude ρ_i and widths t_i, $i \in \{\mathrm{in}, \mathrm{out}, \mathrm{tail}\}$, describing the head and tail regions of the bilayer. The bilayer is assumed to be symmetric, so $\rho_{\mathrm{in}} = \rho_{\mathrm{out}}$ and $t_{\mathrm{in}} = t_{\mathrm{out}}$. The proteins on the inside and outside of the bilayer are described by Gaussian chains, described by an effective radius of gyration R_{g}^{i}, an effective copy number N_c^i, $i \in \{\mathrm{in}, \mathrm{out}\}$, and an excess electron density ρ_c compared with water. The Gaussian chains are proxies for distinct protein patches on the bilayer and can partly overlap with the bilayer, but do not fully penetrate it. To determine the structural parameters, a least-squares fit was performed. The quality of the fit was monitored using the reduced χ^2 function described by

$$\chi^2_{red} = \frac{1}{N - p - 1} \sum_{i=1}^{N} \frac{\left(I_{\mathrm{model}}(q_i) - I_{\mathrm{measured}}(q_i)\right)^2}{\sigma_i^2}, \quad (2)$$

with number of data points N, number of free model parameters p, and intensity standard deviation at data point i σ_i. The fitting was performed using the lsqnonlin function of the MATLAB R2020a (The MathWorks, Natick, MA) Optimization toolbox, and the numerical implementation of the model as in (13). For the fit of the SVs in sucrose buffer, the mean SV size R and the standard deviation of this distribution σ_R was kept constant. The relative ratios of the excess electron densities in the head, tail, and chain regions were also kept constant, all other parameters could vary freely. The first 30 data points were not included in the fit because the signal is influenced by missing background information and instrumental noise in this region. During the fit of the SVs in TRIS buffer, the mean SV size R, the radius standard deviation σ_R, and the excess electron densities ρ_{in}, ρ_{out}, ρ_{tail}, and ρ_c were kept constant at the literature values, all other model parameters were freely varied. Again, the first 30 data points were not included in the fit because the signal is influenced by artifacts of the instrumental setup in this region. The resulting curves as well as the corresponding χ^2_{red} are shown in Fig. 3 *a*, and the resulting fit parameters are tabulated in Table 2. The mean radius of the vesicles including the full bilayer as well as the protein shell on the outside of the vesicle is then calculated by $R_{full} = R + \sqrt{2\pi}(t_{\mathrm{out}} + 0.5t_{\mathrm{tail}} + R_{\mathrm{g}}^{\mathrm{out}})$. This results in $R_{\mathrm{full,sucrose}} = 27.6$ nm and $R_{\mathrm{full,TRIS}} = 27.4$ nm. Note that R_{full} defined in this way describes the maximum radius that would still enclose the largest outer proteins, namely the ATP synthases. Hence, given proper orientations, close contact without vesicle deformation could therefore even happen at distances $d \leq 2R_{\mathrm{full}}$.

In Fig. 3 *b*, the bimodal size distributions obtained from the fits in Fig. 3 *a* are shown. Since the mean radius and standard deviation for the smaller size fraction was fixed, the distributions deviate only in the amplitude. The distributions reveal that the preparation in sucrose buffer contains a larger fraction of small SVs compared with the preparation in TRIS buffer. Reciprocally, the preparation in TRIS buffer contains more of the larger membranous particles. Notably, the size distribution of the larger particles is broader compared with the preparation in sucrose buffer. In Fig. 3, *c* and *d* the electron density profile of the lipid bilayer and protein shells is shown for the

TABLE 2 Parameters resulting from the least-squares fit of the SV samples

Model fit parameter	SV Komorowski et al.	SV Castorph et al.	SV sucrose buffer	SV TRIS buffer	Unit
ρ_{in}, ρ_{out}	46.8	46.8	35.3	46.8	$e^{-}nm^{-3}$
ρ_{tail}	−28.8	−28.8	−40.3	−28.8	$e^{-}nm^{-3}$
t_{in}, t_{out}	1.6	1.79	1.86	1.62	nm
t_{tail}	2.33	2	0.96	1.07	nm
R_g^{in}	2.51	2.86	2.3	1.7	nm
R_g^{out}	4.38	5.3	4.8	5.01	nm
$N_c^{in}/(4\pi(R - D - R_g^{in})^2)$	0.0179	0.0084	0.027	0.04	nm^{-2}
$N_c^{out}/(4\pi(R + R_g^{in})^2)$	0.00136	0.0009	0.00097	0.0009	nm^{-2}
ρ_c	52.1	52.1	40.6	52.1	$e^{-}nm^{-3}$
R	16.95	16.95	16.95	16.95	nm
σ_R	3.92	3.92	3.92	3.92	nm
Amplitude	248.19	248.19	253.9	237.5	Arb. units
R_{large}	277.84	328.58	280.15	297.92	nm
$\sigma_{R,large}$	40.8	82.5	51.7	82.33	nm
$Amplitude_{large}$	0.43	1.22	0.84	0.99	Arb. units
Scale	1.0097	0.0838	0.77	0.095	–
Constant background	0.00109	0.00019	0.0028	-9.66×10^{-6}	1/mm
χ^2_{red}	67.8	4.99	52.19	18.82	

Fit results corresponding to the fits in Fig. 3, with the model described in (13,14). In the model, vesicles are described as polydisperse spherical particles with a mean radius R, standard deviation σ, and amplitude of the Gaussian distribution. To account for contamination by larger membranous particles, a second size distribution with radius R_{large}, width σ_{large} and $amplitude_{large}$ is introduced. The lipid bilayer is described by three Gaussians with amplitudes ρ_i, which describe the excess electron density (compared with water), while $t_i, i \in \{in, out, tail\}$ describe the width of the head and tail regions, respectively. The bilayer is assumed to be symmetric, i.e., $t_{in} = t_{out}$ and $\rho_{in} = \rho_{out}$. The overall thickness of the bilayer is given by $D_b = \sqrt{2\pi}(t_{in} + t_{tail} + t_{out})$. Proteins around the lipid bilayer are described by Gaussian chains with radius of gyration R_g^i, copy number $N_c^i, i \in \{in, out\}$, and the electron density ρ_c. All electron densities are given as the density difference to the buffer solution. The parameters for the SV fractions from previous studies can be found in (14). Confidence intervals for the model parameters found in this study are given in the supporting material.

preparation in sucrose buffer and the preparation in TRIS buffer, respectively. The qualitative shape of the two profiles is very similar.

SV condensates

Following the SAXS measurements of the individual constituent solutions, condensates were formed by mixing SVs and synapsin at different concentrations (11). To this end, a sample series of condensates with different protein/lipid ratios P/L were prepared and measured. The molar concentrations and corresponding P/L values of samples measured in this study are tabulated in Table 3. For these measurements, mainly the preparations of SVs in sucrose buffer were used. Since the background of the sucrose buffer was not properly measured, instead of a combination of TRIS buffer and sucrose buffer, pure TRIS buffer was subtracted as background. The scattered intensities measured on condensates of synapsin and SVs in sucrose buffer at a detector distance of 3 m are shown in Fig. 4 *a*. The curves obtained for a condensate of synapsin and SVs in TRIS buffer are shown in Fig. S2, *a* and *c* in the supporting material. The characteristic modulations of the SVs are still visible in the SV-synapsin-condensate curves, but even for the lowest synapsin concentration $P/L = 1:1118$ a qualitative change of the shape of the scattered intensity is visible. Especially in the low q region, the slope of the curves differ substantially. Next, we pose our analysis on the assumption that the scattered intensity of identical and at least roughly spherical particles in solution is described by $I(q) \propto F(q)S(q)$, where $F(q)$ denotes the particle form factor and $S(q)$ the structure factor. The form factor $F(q) = <|f(\mathbf{q})^2|> = <|\int \rho(\mathbf{r}) \exp(i\mathbf{qr})d\mathbf{r}|^2>$ reflects the size and shape of the scattering particles with $\langle \ldots \rangle$ denoting an ensemble and orientational average. The structure factor $S(q) = <|\sum_n \exp(i\boldsymbol{q}\boldsymbol{r_n})|^2>$ describes the interparticle interactions and correlations. In a dilute sample, there is no interaction between the particles and $S_{dilute} = 1$.

The structure factor $S(q)$ of the vesicle-synapsin condensates can therefore, at least to some approximation, be determined by dividing the intensity I_{SVSyn} measured for SV-synapsin condensates by the intensity curves I_{SV} measured for the dilute vesicle sample. From $S(q)$ we can in turn deduce intervesicle distances and correlations induced by synapsin. The structure factors for the curves presented in Fig. 4 *a* are shown in Fig. 4 *b*. For a P/L of 1:41, it is

TABLE 3 P/L and molar concentrations of the SV-synapsin condensate samples measured in this study

P/L	Synapsin (μM)	SV (nM)
1:41	10.125	60
1:124	6.75	120
1:373	3.375	180
1:1118	1.35	216

For the calculation, 6992 phospholipids per SV were assumed, following (2).

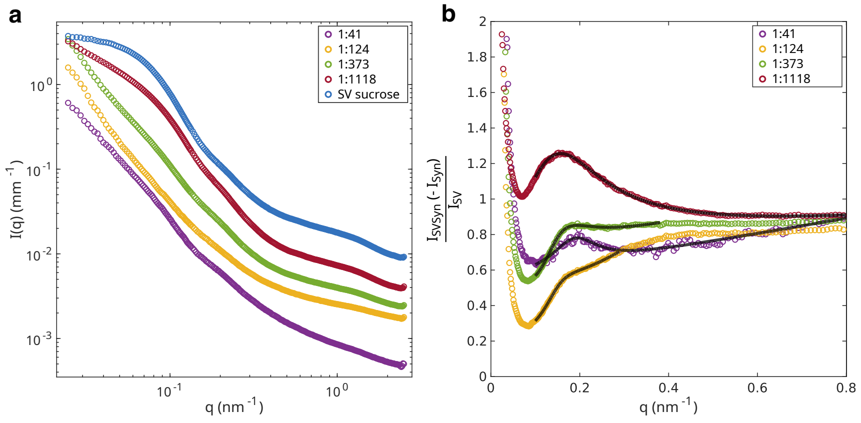

FIGURE 4 (*a*) Comparison of scattering curves for different P/L ratios and molar concentrations for synapsin and SVs as tabulated in Table 3, measured at a detector distance of 3 m. The curves are shifted vertically for clarity. (*b*) Structure factors for different P/L ratios and corresponding least-square fits.

reasonable to assume that there are substantial amounts of free synapsin molecules in the sample, so, for this sample of highest concentration, pure synapsin was subtracted before calculating the structure factor. A comparison of all structure factors with and without the subtraction of synapsin are shown in Fig. S3 in the supporting material. All calculated structure factors show a distinct peak or a shoulder at around $0.15\ \text{nm}^{-1} \le q \le 0.2\ \text{nm}^{-1}$. To determine the exact position of the peaks, an empirical lineshape model was used, given by a (generalized) skewed Cauchy-Lorentz distribution combined with added linear background, written as

$$I_{CL}(q) = \frac{\text{scale}}{\sigma\pi}\left(1 + \frac{(q-\mu)^2}{\sigma^2(1+\lambda(q-\mu))^2}\right)^{-1} + mq + b, \tag{3}$$

where μ is a peak position parameter, σ a parameter for its width, λ a skewness factor, was fitted to the curves. The model parameters determined by the fit are tabulated in Table 4. To rule out that the fitted maxima are misleadingly caused by the missing buffer information, Fig. S2 shows the curve for condensates formed from SVs in TRIS buffer at $P/L = 1:70$, for which the exact buffer background was available. Comparing this curve with the SV sucrose condensate with $P/L = 1:124$ shows a peak at the same q position, corroborating the use of the proxy background.

If we assume the vesicle condensates to be formed by a compact dense fluid of vesicles, the intervesicle distance d measured between the centers of neighboring vesicles would be given by $d = \frac{2\pi}{q^*}$, with q^* denoting the position of the first structure factor maximum. From the peak positions of the experimental curves, which have been accurately determined by the least-squares fits to the empirical lineshape model, we would calculate mean vesicle distances ranging between $d = 33.2$ nm for the tightest condensates at $P/L = 1:41$, and $d = 40.8$ nm for the more loosely bound condensates at $P/L = 1:1118$. Given the fitted radii of SVs, we would then always face a situation with $d < 2R$. The proteins of adjacent SVs would need to penetrate each other and, even for the more loosely bound condensates, SVs would need to deform to be so close. Note that the full radius for the SVs in sucrose, i.e., the radius with protein corona, was measured to 27.6 nm, and even when not taking the patchy outer protein layer into account, the outer bilayer radius 22.8 nm would still be too large for such a close spacing. We therefore conclude that there must be a deviation from a dense compact fluid, putting into question how d is calculated from the position of the first structure factor maximum. Indeed, as we show next, the relationship $d \propto \frac{1}{q^*}$ has a different prefactor for fractal-like aggregation, i.e., when SVs do not form compact aggregates in a condensate. A similar effect in the mismatch between the particle size and the position of the first maximum, was also already reported earlier in other systems of liquid-liquid phase separation, namely in urate oxidase/PEG mixtures (15). Note that the above relationship is not generally valid, and depends on the type of liquid.

TABLE 4 Model fit parameters obtained from least-squares fits to the structure factors of SV-synapsin condensates using a skewed Cauchy-Lorentz distribution and a linear slope

Model fit parameter	1:41	1:124	1:373	1:1118	1:70 (3 m)	1:70 (10 m)
Scale	0.032 ± 0.008	0.032 ± 0.005	0.073 ± 0.006	0.25 ± 0.03	0.006 ± 0.003	0.21 ± 0.05
σ	0.064 ± 0.011	0.07 ± 0.009	0.089 ± 0.006	0.128 ± 0.005	0.022 ± 0.01	0.060 ± 0.009
μ	0.189 ± 0.005	0.173 ± 0.003	0.181 ± 0.002	0.154 ± 0.002	0.172 ± 0.006	0.173 ± 0.004
λ	0.5 ± 1.1	8.5 ± 1.91	8.4 ± 0.9	2.7 ± 0.3	-4.1 ± 11.4	5.8 ± 0.99
m	0.44 ± 0.04	1.7 ± 0.15	0.63 ± 0.08	0.2 ± 0.03	2.4 ± 0.2	8.6 ± 4.8
b	0.54 ± 0.022	0.13 ± 0.03	0.48 ± 0.03	0.61 ± 0.05	0.76 ± 0.04	1.26 ± 0.05

Deviations from spherical symmetric particles and also the type of interparticle potential can lead to deviations (23, 24). For the present case of nearly spherical particles that come into close contact, a deviation can in particular be expected if the assumption of a homogeneous density is lifted. As we show next, we can solve the apparent contradiction if we assume that the SVs form fractal-like condensates with a few contact sites per vesicle rather than a dense vesicle fluid. To obtain the geometry-dependent prefactor in the relationship between intervesicle (next neighbor) distance d and q^*, we turn to a simulation modeling fractal aggregation.

Fractal cluster simulation

Next, we compute structure factors from simulated fractal clusters and analyze how the first maximum of the structure factor depends on the parameters of the fractal clusters, in particular the fractal dimension D. To this end, we use the algorithm developed by Tomchuk et al., which was designed to generate fractal aggregate clusters (25). This stochastic and nonkinetic algorithm uses a hierarchical procedure for the generation of clusters. The description of the fractal clusters is based on the concept of the fractal dimension D, describing the relation between the aggregation number N (number of particles in a cluster) and the radius of gyration R_g by

$$N = k\left(\frac{R_g}{a}\right)^D, \tag{4}$$

where the exponent D denotes the fractal dimension, k is a prefactor, and a is the radius of the particles. The algorithm works as follows: in a hierarchical assembly, two clusters of the same size (same N) are combined to form a larger cluster. For this, the two clusters (denoted by indices 1 and 2) are positioned such that their centers of mass (centroids) have a distance of

$$\Gamma = \sqrt{\frac{M^2}{M_1 M_2}a^2\left(\frac{N}{k}\right)^{2/D} - \frac{M}{M_2}R_{g1}^2 - \frac{M}{M_1}R_{g2}^2}, \tag{5}$$

with masses $M_{1/2}$ and $M = M_1 + M_2$. Thus, both tunable parameters D and k determine the compactness of the clusters by regulating the distances Γ. Using a Monte Carlo implementation, the clusters are rotated around randomly selected axes through the centroid by randomly drawn angles. This rotation is continued until at least one “rigid bond” between the cluster is formed, i.e., at least one touch point but no overlap. For a fast implementation, rotations are calculated using quaternions. The radius of gyration R_g of the resulting cluster is calculated from those of the two constituents according to

$$R_g = \sqrt{\frac{M_1}{M}R_{g1} + \frac{M_2}{M}R_{g2} + \frac{M_1 M_2}{M^2}\Gamma^2}. \tag{6}$$

The steps described above are iterated. In this way a cluster is generated by a hierarchical assembly. The iterative growth of the cluster with correspondingly increasing R_g is initialized with $R_g = \sqrt{3/5}R$, the obvious value for an isolated spherical particle. Combining two single-particle clusters to a dimer, the initial distance between the centroids is $\Gamma_{\mathrm{init}} = R_1 + R_2$, R_g given by Eq. 6. In the same way, a second dimer is created. Next, random orientations are attributed to the dimers, and two dimers are combined to one tetramer according to the steps described above.

Fig. 5 shows the result of the cluster simulation and the corresponding structure factors $S(q) = <\left|\sum_n \exp(i\boldsymbol{q}\boldsymbol{r_n})\right|^2>$, calculated by averaging over a large ensemble of simulated clusters. In Fig. 5 *a*, $S(Q)$ is shown for different D, at constant prefactor $k = 1.2$. Note that the momentum transfer is measured in natural units $Q = qa$. On log-log plot, a linear decay is observed in the Guinier regime ($Q = qa < 1$), followed with damped oscillation around one, the asymptotic limit for large Q. The functional form of $S(Q)$ is then further analyzed, in view of Fig. 5 *b* the power law exponent γ describing the initial decay of $S(Q)$, and Fig. 5 *c* the position of the first structure factor maximum Q^*. The exponent γ is determined by a power law fit in the range $Q \approx 0.1$ and $Q \approx 0.6$. The resulting linear dependence D confirms the relationship $\gamma = -D$ expected for diffraction from fractal geometries, as already shown in (25). To obtain the values Q^* shown in Fig. 5 *c*, the position of the first maximum in the structure factor was identified by a peakfinder algorithm. Since there is less noise in the simulation compared with the experimental data, and the sampling can be adapted, the accurate determination of the peak is rather straightforward and does not require least-squares fitting. Alternatively, one can determine the peak position by fitting the data to an empirical lineshape function such as in the experimental case. To this end, a Cauchy-Lorentz distribution turned out to be well suited, with

$$S_{CL}(Q) = \frac{1}{\pi}\left(\frac{\sigma}{(Q - Q^*)^2 + \sigma^2}\right),$$

with the position of the first maximum Q^* and the width parameter σ (halfwidth at half-maximum). Q^* is found to increase with decreasing D, confirming the hypothesis that the prefactor in $d \propto 1/q^*$ is D dependent. Note that a constant value $Q^* = \pi$ corresponds to $q^* = 2\pi/(d = 2a)$, i.e., the “classical” relationship. Next, Fig. 5 *d* shows structure factors for different values of the fractal prefactor k at a fixed value of $D = 2$. In addition to D, k also affects the shape of the structure factor curve including a shift of the position of the first local maxima and minima. Finally, as shown in Fig. 5 *e*, we included polydispersity of the particles in the simulation, governed by a polydispersity parameter σ_P. Particles that previously were monodisperse

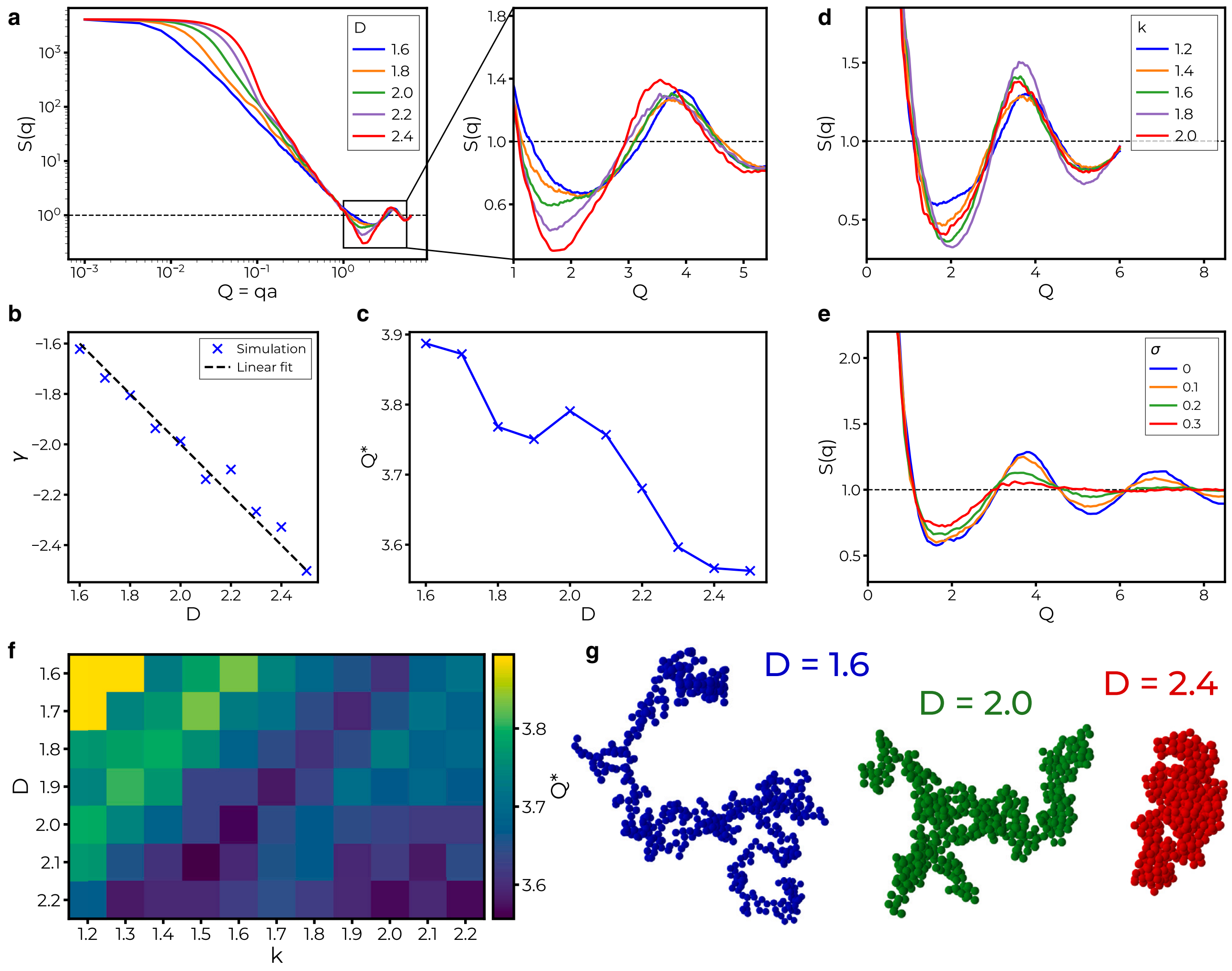

FIGURE 5 Simulation of fractal clusters using the approach from Tomchuk et al. (25). (*a*) Structure factors are shown from clusters with fixed $k = 1.2$ and different values of the fractal dimension D. Curves are plotted on the scale $Q = qa$ which is independent of the mean particle radius a. The decay of the SF in the Guinier regime changes with D. Zooming into the region of the first correlation shell reveals a change of shape and position of the local maxima. (*b*) By linear fitting the slopes of the SF in the Guinier regime from $Q \approx 0.1$ to $Q \approx 0.6$ and plotting against D, it can be seen that the slope in this regime is correlated to the fractal dimension by the relation $\gamma \approx -D$. (*c*) Plotting the peak position of the first correlation shell Q^* against D reveals a negative correlation. The observed values of Q^* does not coincide with the classical peak position for a dense hard core fluid of $Q^* = \pi$. However, for high D, which represent the clusters with compact structures, Q^* seems to converge to this value. (*d*) SF of simulated clusters at fixed $D = 2$ for varying k. (*e*) The simulation can be extended by introducing polydisperse particles, wherein particle radii are randomly generated following a log-normal distribution. The parameter σ approximately gives the standard deviation of the distribution and controls the level of polydispersity. SF values of polydisperse cluster are shown for fixed $k = 1.2, D = 2$ and different σ. With increasing σ, that is with higher polydispersity, the amplitude of the extrema decreases. (*f*) First peak position Q^* in dependence of both fractal parameters D and k. A pattern combining an oscillation with a linear decay is obtained. For compact clusters (high D and k), the peak position decreases. (*g*) Visual representation of three clusters created at different values of the fractal dimension D (monodisperse, $k = 1.2$). By tuning D, either elongated and fractal or dense and compact clusters can be generated.

(radius $a = 1$) are now drawn from a log-normal distribution

$$p(a) = \frac{1}{a\sigma_P\sqrt{2\pi}} \exp\left(-\frac{(\ln a - \mu)^2}{2\sigma_P^2}\right) \tag{7}$$

with $\mu = 1$ fixed, and σ_P varied as indicated in the legend. As expected, an increasing σ_P results in a damping of the higher maxima. The four examples shown in Fig. 5 *e* are computed at varied $\sigma_P = 0, 0.1, 0.2, 0.3$ for fixed values of $D = 2$ and $k = 1.2$. At $\sigma = 0.3$, the first maximum reduces to a mere shoulder. For completeness, Fig. 5 *f* then shows the full dependence of $Q^*(D, k)$, i.e., as a function of both the fractal dimension D and the prefactor of k. Again, the classical value for $Q^* = \pi$ is nowhere reached. However, the values decrease and approach the value π from above when clusters become more and more compact for larger D and k. For visual inspection, examples of clusters are shown in Fig. 5 *g*, for three different values of D. With increasing D, the structure of the cluster changes

from an elongated and fractal to a more dense and compact structure.

Given the insights gained from the simulation, we can turn to the experimental data again, in view of matching experimental data and simulated structure factor. In other words, we try to find simulation parameters for which clusters exhibit similar properties as the measured SV-synapsin condensates. Fig. 6 shows the data recorded at $P/L = 1:373$, and an overlay with the scaled structure factor for clusters simulated with the model above. From Fig. 6 *a*, we can infer that the data at low q decays with a power-law of $\gamma_{1:373} = -2.43$ (see linear fit on log-log scale). Accordingly, we fixed $D = 2.4$ for the SF simulation. For values of $k = 0.6$ and $\sigma = 0.3$, the overlay of simulation and data in Fig. 6 *b* show reasonable agreement for the power-law decay, the first minimum and the upturn to the shoulder at Q^*, while the curves do not overlap at higher $Q > Q^*$. For this comparison, the experimental data had to be scaled to the natural units $Q = qa$, as in the simulation. Here, for given D and k, a value of $a = 20$ nm or correspondingly $d = 2a = 40$ nm yield reasonable agreement. Concerning the polydispersity, smaller values of σ result in more pronounced modulations, which are not observed for this data set, see the simulated curve for $\sigma = 0.23$ in Fig. 6 *b*.

Note, however, that the comparison of simulated and experimental structure factor tends to higher d when assuming a lower value of D, and hence the value of $d =$ 40 nm must be rather regarded as a lower bound. To this end, we have to question the validity of fixing D from γ, which was fitted in a range of $0.5 \leq Q \leq 0.76$. We have to keep in mind that the division by the form factor is only approximative, since SVs are not spherically symmetric and identical particles. Moreover, regarding the cluster simulations, the structure factor in this range is also influenced by k, so that the relation $\gamma = -D$ is not necessarily valid. In the comparison between data and simulations, smaller D would result in larger distances $d > 40$ nm, which seems more realistic. At $d \simeq 43 - 48$ nm, the outer protein layers would only partially overlap or interpenetrate, and synapsin could also partition into the intervesicle gap.

DISCUSSION

Given the significant influence of D and k on the position of the peak maximum, and hence the scaling factor needed for overlap of experimental and simulated curve, the resulting intervesicle spacing d can be shifted within a certain range, with $d > 40$ nm as a lower bound. Larger and more realistic values of d in view of the SV protein corona and synapsin layer, can be obtained if one assumes lower $D < 2.4$, relaxing the requirement that D is fixed from the initial decay of the structure factor, which can be justified in view of several unwarranted idealizations (nonidentical particles, q range, contamination by larger membranous particles). Moreover, the fractal cluster model may be an oversimplification altogether and could be replaced by a full model and Monte Carlo simulations based on modeled interaction forces in the future. Notwithstanding these limitations, we can use this simple fractal model to show that the position of the first structure factor maximum does not lie at $Q^* = \frac{2\pi}{2a} = \pi$, but takes on significantly higher values around 3.6–3.9. Therefore one would "falsely" deduce too short a distance, when analyzed by the classic relationship. Due to the nonuniqueness in the comparison between data and model (notably the uncertainty in determining the parameters D and k), a range of d values is possible. Despite this uncertainty, the entire range of which d can be shifted, indicates surprisingly small intervesicle contacts at least of some vesicles induced by synapsin. These tight contacts would involve partial interpenetration of outer membrane proteins to which synapsin must associate as well. Importantly, and with very little doubt we can infer that the vesicle synapsin condensates do not exhibit a structure of a classical compact liquid, but exhibits a morphology that is not dense but has many open spaces and passages. In view of the small

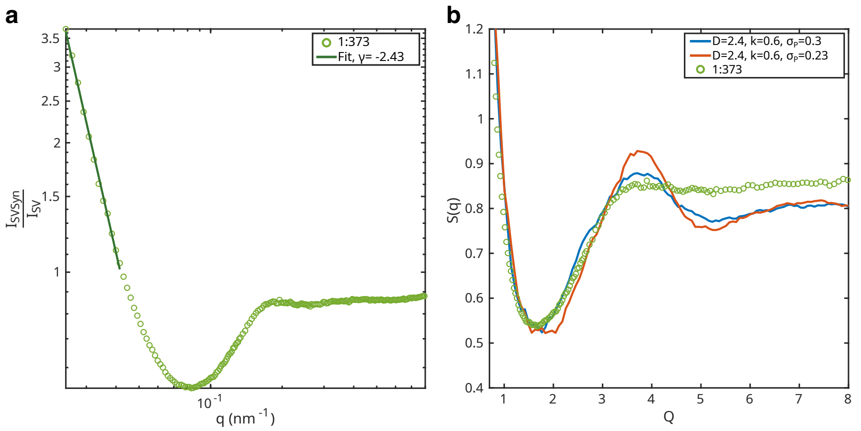

FIGURE 6 (*a*) Structure factor measured on the sample containing SV and synapsin with a $P/L = 1:373$. The slope $\gamma = -2.43$ was determined by a linear fit in log-log for $0.025\ \mathrm{nm}^{-1} \leq q \leq 0.038\ \mathrm{nm}^{-1}$. (*b*) Comparison of structure factors obtained on simulated clusters with $D = 2.4$, $k = 0.6$, and $\sigma = 0.23$ and $\sigma = 0.3$, respectively, to the structure factor of the sample with $P/L = 1:373$. The experimental structure factor was scaled on the *x* axis with a factor of 20 nm. The simulated structure factor was shifted in intensity by a factor of − 0.2.

size of the condensates (or clusters in the simulations), one may not embrace the fractal concept or terminology, but alternative model formulations would also need to account for the noncompact morphology. The significance that this morphology could bear for biological function, may relate to enhanced accessibility for metabolites, diffusion, and all transport processes in a synaptic pool. Note, however, that electron micrographs of synaptic pools do not show particularly short d, and neither give the impression of a fractal cluster. Hence, this may also point to the limits of SV-synapsin as an in vitro model for synaptic pools, and the role of further proteins, mechanisms, or processes underlying the organization of pools in the synapse. From the perspective of colloidal aggregation, an efficient repulsive interaction would be required to shift from fractal to more compact aggregate morphology. At the same time, a higher mobility of SV and synapsin in the condensate would also help to relax a fractal morphology to a more compact fluid, which is not seen here. Instead a noncompact morphology is found, pointing to a rather strong attraction similar to a "hit and stick" mechanism as in diffusion-limited aggregation (26). A network structure of synapsin and SVs with low subdiffusive mobility as reported in (27) based on single-molecule tracking is well in line with this scenario.

CONCLUSION

In summary, we have successfully measured pure synapsin solution, with indication of partially unfolded structure. For the pure SV solutions (suspensions) by SAXS, we found no significant structural changes in the two different buffers investigated. We then showed that SAXS is also well suited to investigate the condensate formation of SVs and synapsin, complementing cryo-EM and optical microscopy by a high-resolution room temperature technique, albeit relying on an ensemble average and indirect model-based analysis. As a main focus of this work, we then investigated condensate structure by different P/L. By analysis of the structure factor and cluster simulations, we could shed light on the morphology of SV-synapsin condensates and intervesicle distances. Not only the shift of the maximum, but the entire shape of the structure factor indicate that the condensates cannot be explained by a compact fluid structure, but rather exhibits a fractal geometry of more loosely associated vesicles. Note that, due to the limited size of the condensates, the fractal geometry, however, does not imply a true scale invariance, but simply a more noncompact and open structure. The parameter D is in this case to be regarded rather as a morphology parameter than a fractal dimension in the classical sense. Together with the parameter k it also describes the statistical distribution of vesicle contacts. Most importantly, vesicles do not exhibit constant density in the condensate as in a compact fluid, but rather a loosely bound aggregate with open interior spaces, similar to the geometry of the simulated clusters. This may bear functional consequences by favoring transport and exchange of metabolites.

ACKNOWLEDGMENTS

We thank Reinhard Jahn for his advice and support of the project, in particular in view of synaptic vesicles purification, Karlo Komorowski for sharing expertise in vesicle preparation, Markus Osterhoff for data management support, Silvio Rizzoli for discussion and support, and the entire SFB1286 *Quantitative Synaptology* for a stimulating collaborative research environment. We acknowledge DFG for funding through grant SFB1286/A2.

AUTHOR CONTRIBUTIONS

T.S., D.M., C.N., and J.A. designed the research. C.N., J.A., and L.M. carried out the synchrotron experiments. R.J., M.G., C.H., and D.M. prepared/purified the samples and contributed biochemical expertise. C.N. and J.A. analyzed the data, J.F. and C.N. carried out the simulations. T.S. provided advice for analysis and simulation. C.N. and T.S. wrote the first draft, which was discussed and iterated between all authors.

DECLARATION OF INTERESTS

The authors declare no competing interests.

SUPPORTING MATERIAL

Supporting material can be found online at https://doi.org/10.1016/j.bpj.2024.11.004.

REFERENCES

1. Rizzoli, S. O., and W. J. Betz. 2005. Synaptic vesicle pools. *Nat. Rev. Neurosci.* 6:57–69.
2. Takamori, S., M. Holt, ..., R. Jahn. 2006. Molecular Anatomy of a Trafficking Organelle. *Cell.* 127:831–846. https://www.sciencedirect.com/science/article/pii/S0092867406014000.
3. Birks, R., and F. C. MacIntosh. 1961. Acetylcholine metabolism of a sympathetic ganlion. *Can. J. Biochem. Physiol.* 39:787–827.
4. Hilfiker, S., V. A. Pieribone, ..., P. Greengard. 1999. Synapsins as regulators of neurotransmitter releas. *Philos. Trans. R. Soc. Lond. B Biol. Sci.* 354:269–279.
5. Wilhelm, B. G., S. Mandad, ..., S. O. Rizzoli. 2014. Composition of isolated synaptic boutons reveals the amounts of vesicle trafficking proteins. *Science.* 344:1023–1028. https://doi.org/10.1126/science.1252884.
6. Zhang, M., and G. J. Augustine. 2021. Synapsins and the Synaptic Vesicle Reserve Pool: Floats or Anchors? *Cells.* 10:658.
7. Benfenati, F., M. Bähler, ..., P. Greengard. 1989. Interactions of synapsin I with small synaptic vesicles: distinct sites in synapsin I bind to vesicle phospholipids and vesicle proteins. *J. Cell Biol.* 108:1863–1872.
8. Cheetham, J. J., S. Hilfiker, ..., A. J. Czernik. 2001. Identification of synapsin I peptides that insert into lipid membranes. *Biochem. J.* 354:57–66. https://doi.org/10.1042/bj3540057.
9. Milovanovic, D., Y. Wu, ..., P. De Camilli. 2018. A liquid phase of synapsin and lipid vesicles. *Science.* 361:604–607.
10. Hoffmann, C., R. Sansevrino, ..., D. Milovanovic. 2021. Synapsin Condensates Recruit alpha-Synuclein. *J. Mol. Biol.* 433:166961:phase Separation in Biology and Disease: The Next Chapter. https://www.sciencedirect.com/science/article/pii/S0022283621001625.

11. Alfken, J., C. Neuhaus, T. Salditt…, 2023. Vesicle condensation induced by synapsin: condensate size, geometry, and vesicle shape deformations. *Eur. Phys. J. E.* 47. https://doi.org/10.1140/epje/s10189-023-00404-5.

12. Sansevrino, R., C. Hoffmann, and D. Milovanovic. 2023. Condensate biology of synaptic vesicle clusters. *Trends Neurosci.* 46:293–306. https://www.sciencedirect.com/science/article/pii/S0166223623000152.

13. Castorph, S., D. Riedel, …, T. Salditt. 2010. Structure Parameters of Synaptic Vesicles Quantified by Small-Angle X-Ray Scattering. *Biophys. J.* 98:1200–1208. https://www.sciencedirect.com/science/article/pii/S0006349509060913.

14. Komorowski, K., J. Preobraschenski, …, T. Salditt. 2022. Neurotransmitter uptake of synaptic vesicles studied by X-ray diffraction. *Eur. Biophys. J.* 51:465–482. https://doi.org/10.1007/s00249-022-01609-w.

15. Vivares, D., and F. Bonnete. 2004. Liquid-Liquid Phase Separations in Urate Oxidase/PEG Mixtures: Characterization and Implications for Protein Crystallization. *J. Phys. Chem. B.* 108:6498–6507.

16. Fold, A. 2023. Synapsin 1. https://alphafold.ebi.ac.uk/entry/P17600.

17. Narayanan, T., M. Sztucki, …, P. Boesecke. 2018. A multipurpose instrument for time-resolved ultra-small-angle and coherent X-ray scattering. *J. Appl. Crystallogr.* 51:1511–1524. https://doi.org/10.1107/S1600576718012748.

18. Narayanan, T., M. Sztucki, …, P. Boesecke. 2022. Performance of the time-resolved ultra-small-angle X-ray scattering beamline with the Extremely Brilliant Source. *J. Appl. Crystallogr.* 55:98–111. https://doi.org/10.1107/S1600576721012693.

19. Sztucki, M. 2021. SAXSutilities2: a graphical user interface for processing and analysis of Small-Angle X-ray Scattering data. https://doi.org/10.5281/zenodo.5825707.

20. pyMOLwiki. 2019. Radius of gyration. https://pymolwiki.org/index.php/Radius_of_gyration.

21. Schrödinger, L., and W. DeLano. PyMOL. http://www.pymol.org/pymol.

22. UniProt. 1990. P17600:SYN1 HUMAN. https://www.uniprot.org/uniprotkb/P17600/history.

23. Tago, Y. 1973. Spatial structure and thermodynamic properties of a classical fluid of hard spheres with attractive square wells. *J. Chem. Phys.* 58:2096–2109. https://doi.org/10.1063/1.1679476.

24. Gazzillo, D., and A. Giacometti. 2000. Structure factors for the simplest solvable model of polydisperse colloidal fluids with surface adhesion. *J. Chem. Phys.* 113:9837–9848.

25. Tomchuk, O. V., M. V. Avdeev, and L. A. Bulavin. 2020. Modeling fractal aggregates of polydisperse particles with tunable dimension. *Colloids Surf. A Physicochem. Eng. Asp.* 605:125331. https://doi.org/10.1016/j.colsurfa.2020.125331.

26. Witten, T. A., and L. M. Sander. 1981. Diffusion-limited aggregation, a kinetic critical phenomenon. *Phys. Rev. Lett.* 47:1400–1403.

27. Hoffmann, C., J. Rentsch, …, D. Milovanovic. 2023. Synapsin condensation controls synaptic vesicle sequestering and dynamics. *Nat. Commun.* 14:6730.

Biophysical Journal, Volume *123*

Supplemental information

Morphology and intervesicle distances in condensates of synaptic vesicles and synapsin

Charlotte Neuhaus, Jette Alfken, Jakob Frost, Lauren Matthews, Christian Hoffmann, Marcelo Ganzella, Dragomir Milovanovic, and Tim Salditt

Synaptic vesicles

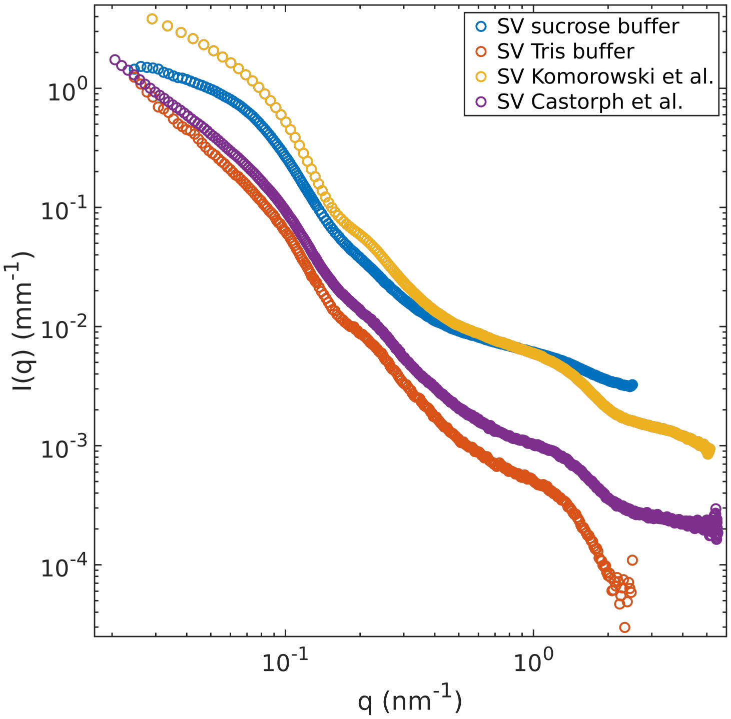

Figure S1: Comparison of different scattering curves obtained on synaptic vesicles in previous studies by Komorowski et al. (yellow) (1) and Castorph et al. (violet) (2) and in this study (blue,red). Especially in the intermediate q-range, all curves exhibit a similar functional form sharing characteristic modulations. The deviations in the small q-range are caused by the different amount of contamination by larger membranous particles.

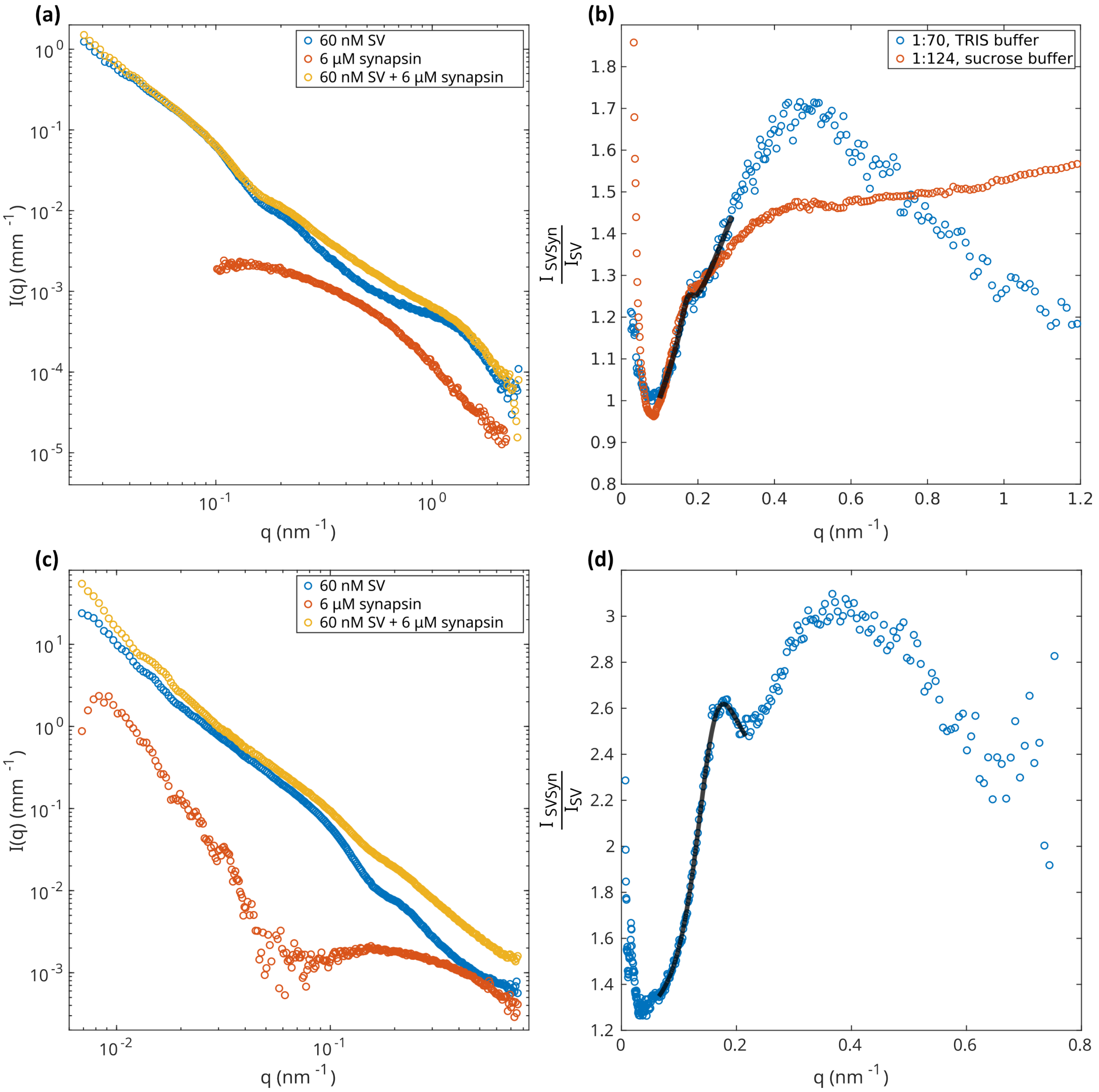

Figure S2: Scattered intensities and corresponding structure factors for measurements on SV-synapsin condensates in TRIS buffer. (a) Scattered intensities of samples containing 60 nm SVs in TRIS buffer and 6 μM synapsin, corresponding to a $P/L = 1:70$, measured at a detector distance of 3 m and an acquisition time of 0.1 s. (b) Structure factor a of the scattering curves shown in (a) (blue) and least-square fit with the distribution introduced in Eq. 3 compared to the structure factor of the sample with $P/L = 1:124$ previously shown in Fig. 4 (b)(red). For a better comparison of the structure factors, the 1:124 curve was vertically shifted. (c) Scattered intensity of a sample with $P/L = 1:70$ measured at a detector distance of 10 m with an acquisition time of 0.1 s. (d) Structure factor obtained from the scattering curves in (c) and least square fit with distribution introduced in Eq. 3.

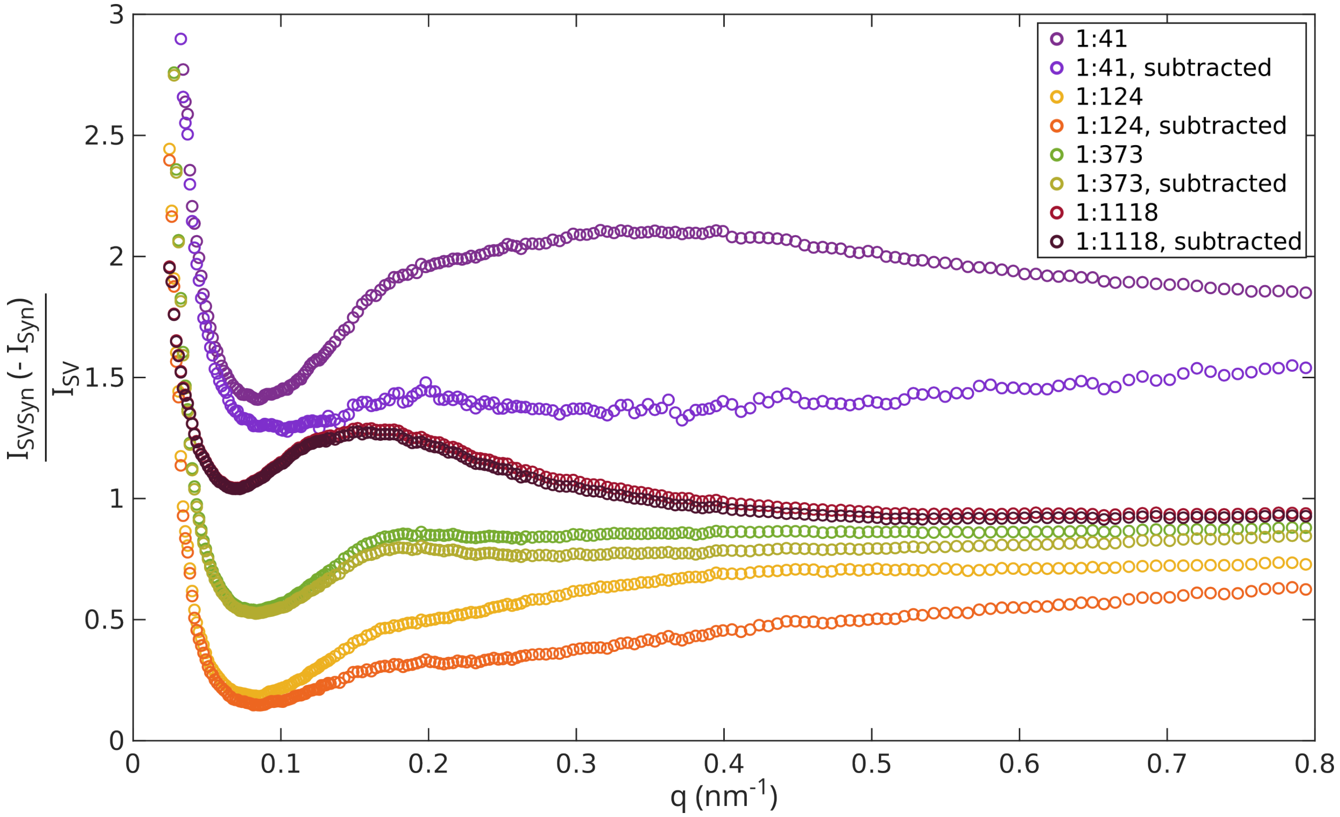

Figure S3: Comparison of structure factors condensates of SVs in sucrose and synapsin with and without the subtraction of synapsin, the curves are shifted vertically for clarity. The structure factors are calculated as described in the result section. The structure factor for a $P/L = 1 : 41$ significantly changes with the subtraction of synapsin and a peak that was previously not visible emerges. For the lower P/Ls the change becomes less significant and decreases with a decreasing P/L.

Error estimation

The fitting errors and covariance matrices for the nonlinear least-square fits shown in Fig. 3 were determined as described in (1). Onehundred pseudo-realizations of the 'experimental' data were generated from randomly drawn values from a normal distribution around each measured data point. The standard derivation of the distribution was described by the experimental errors. By fitting the synaptic vesicle model to the generated data, a parameter vector was obtained, which is then used to calculate the fit errors for each model parameter as well as the covariance matrix. The resulting model parameters and corresponding fit errors are tablated in Tab. S1, the correlation matrices for SVs in sucrose and SVs in TRIS buffer are tabulated in Tab. S2 and S3, respectively. An explanation of the parameters of the covariance matrix is given in Tab. S4.

Model fit parameter	SV sucrose buffer	SV TRIS buffer	Unit
$\rho_{\text{in}}, \rho_{\text{out}}$	35.5 ± 0.3	46.8	e^- nm^{-3}
ρ_{tail}	-40.14 ± 0.3	-28.8	e^- nm^{-3}
$t_{\text{in}}, t_{\text{out}}$	1.86 ± 0.02	1.69 ± 0.03	nm
t_{tail}	0.97 ± 0.02	1.07 ± 0.03	nm
R_g^{in}	2.28 ± 0.04	1.87 ± 0.1	nm
R_g^{out}	4.8 ± 0.05	5.25 ± 0.12	nm
$N_c^{\text{in}}/(4\pi(R-D-R_g^{\text{in}})^2)$	0.028 ± 0.0012	0.031 ± 0.003	nm^{-2}
$N_c^{\text{out}}/(4\pi(R+R_g^{\text{in}})^2)$	0.00096 ± 0.0001	0.0008 ± 0.0001	nm^{-2}
ρ_c	40.7 ± 0.03	52.1	e^- nm^{-3}
R	16.95	16.95	nm
σ_{R}	3.92	3.92	nm
Amplitude	242.7 ± 3.2	232.6 ± 1.6	Arb. units
R_{large}	179.4 ± 6.6	261.6 ± 6.2	nm
$\sigma_{\text{R,large}}$	46.2 ± 2.3	78.6 ± 2.3	nm
$\text{Amplitude}_{\text{large}}$	1.91 ± 0.11	1.25 ± 0.05	Arb. units
Scale	0.79 ± 0.02	0.091 ± 0.004	-
Constant background	0.0028 ± 0.0001	$-3.6 \cdot 10^{-6} \pm 6 \cdot 10^{-6}$	1/mm

Table S1: Model parameters and corresponding fit errors resulting from the least-squares fit of the N=100 generated pseudo experimental data using the synaptic vesicle model. The fitting was performed as described in the results section. The maximum number of iterations was set to 500.

	P_1	P_2	P_3	P_4	P_5	P_6	P_7	P_8	P_9	P_{10}	P_{11}	P_{12}	P_{13}
P_1	1	-0.91	0.96	-0.66	-0.57	0.76	-0.06	0.75	0.53	-0.22	0.17	0.21	0.16
P_2	-0.91	1	-0.98	0.8	0.36	-0.55	0.06	-0.86	-0.63	0.27	-0.2	-0.28	-0.29
P_3	0.96	-0.98	1	-0.74	-0.45	0.66	-0.05	0.83	0.64	-0.27	0.21	0.27	0.29
P_4	-0.66	0.8	-0.74	1	0.25	-0.25	0.03	-0.98	-0.55	0.12	-0.08	-0.15	-0.23
P_5	-0.57	0.36	-0.45	0.25	1	-0.7	-0.49	-0.34	0.09	-0.15	0.04	0.18	0.36
P_6	0.76	-0.55	0.66	-0.25	-0.7	1	-0.09	0.38	0.11	-0.17	0.14	0.13	-0.21
P_7	-0.06	0.06	-0.05	0.03	-0.49	-0.09	1	-0.01	0.07	0.27	-0.08	-0.29	0.13
P_8	0.75	-0.86	0.83	-0.98	-0.34	0.38	-0.01	1	0.59	-0.14	0.1	0.15	0.25
P_9	0.53	-0.63	0.64	-0.55	0.09	0.11	0.07	0.59	1	-0.31	0.3	0.31	0.91
P_{10}	-0.22	0.27	-0.27	0.12	-0.15	-0.17	0.27	-0.14	-0.31	1	-0.97	-0.95	-0.26
P_{11}	0.17	-0.2	0.21	-0.08	0.04	0.14	-0.08	0.1	0.3	-0.97	1	0.91	0.26
P_{12}	0.21	-0.28	0.27	-0.15	0.18	0.13	-0.29	0.15	0.31	-0.95	0.91	1	0.25
P_{13}	0.16	-0.29	0.29	-0.23	0.36	-0.21	0.13	0.25	0.91	-0.26	0.26	0.25	1

Table S2: Correlation matrix for the varied fit parameters obtained for SVs in sucrose buffer.

	P_1	P_2	P_3	P_4	P_5	P_6	P_7	P_8	P_9	P_{10}	P_{11}	P_{12}
P_1	1	-0.87	0.96	-0.7	-0.93	0.85	-0.61	0.8	0.96	-0.77	0.76	0.73
P_2	-0.87	1	-0.95	0.8	0.7	-0.67	0.44	-0.88	-0.82	0.76	-0.65	-0.72
P_3	0.96	-0.95	1	-0.77	-0.86	0.81	-0.56	0.89	0.94	-0.8	0.74	0.75
P_4	-0.7	0.8	-0.77	1	0.53	-0.47	0.48	-0.96	-0.75	0.72	-0.67	-0.68
P_5	-0.93	0.7	-0.86	0.53	1	-0.9	0.53	-0.66	-0.93	0.68	-0.74	-0.65
P_6	0.85	-0.67	0.81	-0.47	-0.9	1	-0.56	0.61	0.9	-0.61	0.62	0.61
P_7	-0.61	0.44	-0.56	0.48	0.53	-0.56	1	-0.51	-0.63	0.58	-0.53	-0.61
P_8	0.8	-0.88	0.89	-0.96	-0.66	0.61	-0.51	1	0.83	-0.77	0.7	0.71
P_9	0.96	-0.82	0.94	-0.75	-0.93	0.9	-0.63	0.83	1	-0.78	0.77	0.75
P_{10}	-0.77	0.76	-0.8	0.72	0.68	-0.61	0.58	-0.77	-0.78	1	-0.94	-0.89
P_{11}	0.76	-0.65	0.74	-0.67	-0.74	0.62	-0.53	0.7	0.77	-0.94	1	0.82
P_{12}	0.73	-0.72	0.75	-0.68	-0.65	0.61	-0.61	0.71	0.75	-0.89	0.82	1

Table S3: Correlation matrix for the varied fit parameters obtained for SVs in TRIS buffer.

P_1	$D/2$, half thickness of the shell
P_2	$N_c^{\text{out}}/(4\pi(R + R_g^{\text{in}})^2)$
P_3	R_g^{out}
P_4	$N_c^{\text{in}}/(4\pi(R - D - R_g^{\text{in}})^2)$
P_5	Scale
P_6	Constant background
P_7	Amplitude
P_8	R_g^{out}
P_9	Fraction $t_{\text{headgroup}}/D$
P_{10}	R_{large}
P_{11}	$\text{Amplitude}_{\text{large}}$
P_{12}	R_{large}
P_{13}	excess scattering of sucrose buffer (compared to water)

Table S4: Explanation of the fit parameters P_i in the correlation matrix.

Lipid Vesicles

Additional to SVs, condensates of lipid vesicles and synapsin were measured. Figure S4 (a) shows the scattered intensities of samples containing 6 μM synapsin, 1.56 mM LV4 and condensates thereof with $P/L = 1 : 260$ measured at a sample distance of 3 m. For the pure LV4 sample, a curve measured at a concentration of 5 mM LV4 was used and the intensity was scaled to fit the intensity of a 1.56 mM LV4 sample.

To obtain more quantitative information on the vesicles, a model for the scattered intensity is fitted to the data. For this fit, the model described in (3, 4) was used. In the model, the vesicles are described as polydisperse spherical particles with a Gaussian distributed polydispersity with mean radius R and width σ_R. The radius is described as the radius to the center of the lipid bilayer. The radial electron density profile of the lipid bilayer is described by three Gaussians as

$$\rho(r) = \sum_{i=1}^{3} \rho_i \exp\left(\frac{-(z-z_i)^2}{2t_i^2}\right), \tag{1}$$

representing the head- and tail-regions of the lipids, with relative electron density ρ_i, position z_i and width t_i. The profile is assumed to be symmetric, so $\rho_\mathrm{h} = \rho_\mathrm{h,in} = \rho_\mathrm{h,out}$, $\sigma_\mathrm{h} = \sigma_\mathrm{h,in} = \sigma_\mathrm{h,out}$ and $z_\mathrm{h} = z_\mathrm{h,in} = -z_\mathrm{h,out}$. The position of the tail-region is fixed at $z_\mathrm{t} = 0$ and the relative electron density at $\rho_\mathrm{t} = -1$, all other model parameters were freely varied. A powerlaw-corrected background described by $I_\mathrm{background}(q) = c_2\, q^{-c_3} + c_4$ was added to the model intensity. The last 330 points were not included in the fitting process. To fit the model to the data a least squares fit with the a reduced χ^2 cost function (see Eq. 2) was performed. For fitting, the lsqnonlin-function of the MATLAB R2020a Optimization toolbox was used, for numerical implementation of the model was performed as described in (5). The resulting model fit curve and the resulting electron density profile are shown in Fig. S4 (a), the corresponding model parameters are tabulated in Tab S5.

The structure factor calculated from the scattering curves in (a) are shown in (b) and (c). While in (c) the intensity of synapsin is subtracted form the intensity of the cluster before the division, no subtraction was performed in (b). Both structure factors show a peak at $q \approx 0.3\,\mathrm{nm}^{-1}$, to determine the exact peak position, a skewed Cauchy-Lorentz distribution (see Eq. 3) was fitted to the data. The resulting model parameters are shown in Tab. S6. Figure S4 (d) shows the scattering curves for samples containing 6 μM synapsin, 11 mM LV4 and condensates thereof measured at a sample distance of 10 m. The structure factors without and with subtraction of synapsin are shown in (e) and (f) respectively. Again, a skewed Cauchy-Lorentz distribution was fitted to the structure factors, the resulting model parameters are shown in Tab. S6.

Model fit parameter	value
t_h	0.34
t_t	0.74
ρ_h	1.42
ρ_t	1 (fixed)
z_h	±1.99
z_t	0 (fixed)
R (nm)	13.85
σ_R	8.1
scale	$1.3 \cdot 10^{-6}$
c_2	$7.4 \cdot 10^{-9}$
c_3	5.2
c_4	$1.6 \cdot 10^{-4}$

Table S5: Model parameters for the least-squares fit of the scattered intensity of 5 mM LV4, shown in Fig. S4 (a) using the model described in (4). The vesicles are described as Gaussian distributed polydisperse particles with a mean radius R and a width σ_R. The electron density profile is described by three Gaussians with amplitude (relative electron densities) ρ_i, width t_i and position $z_i, i \in \{h_{in}, h_{out}, t\}$. The profile is assumed to be symmetric. Additional background was described by a powerlaw correction $I_{background} = c_2 q^{-c_3} + c_4$.

Model fit parameter	3 m (Fig. S4 (b)) no subtraction	3 m, (fig. S4 (c)) synapsin subtracted	10 m (fig. S4 (e)) no subtraction	10 m (fig. S4 (f)) synapsin subtracted
σ	0.119 ± 0.003	0.121 ± 0.004	0.16 ± 0.06	0.20 ± 0.02
μ	0.313 ± 0.003	0.315 ± 0.003	0.299 ± 0.003	0.142 ± 0.007
λ	0.8 ± 0.11	0.66 ± 0.16	0.78 ± 0.092	0.3 ± 0.5
scale	10.82 ± 0.18	10.96 ± 0.22	2.26 ± 0.08	0.73 ± 0.05

Table S6: Parameters resulting from the least squares fits to the calculated LV4-synapsin structure factors, shown in Fig. S4 using a skewed Cauchy-Lorentz distribution (Eq. 3). σ describes the HWHM of the distribution, μ the position of the peak and λ the skewness of the distribution.

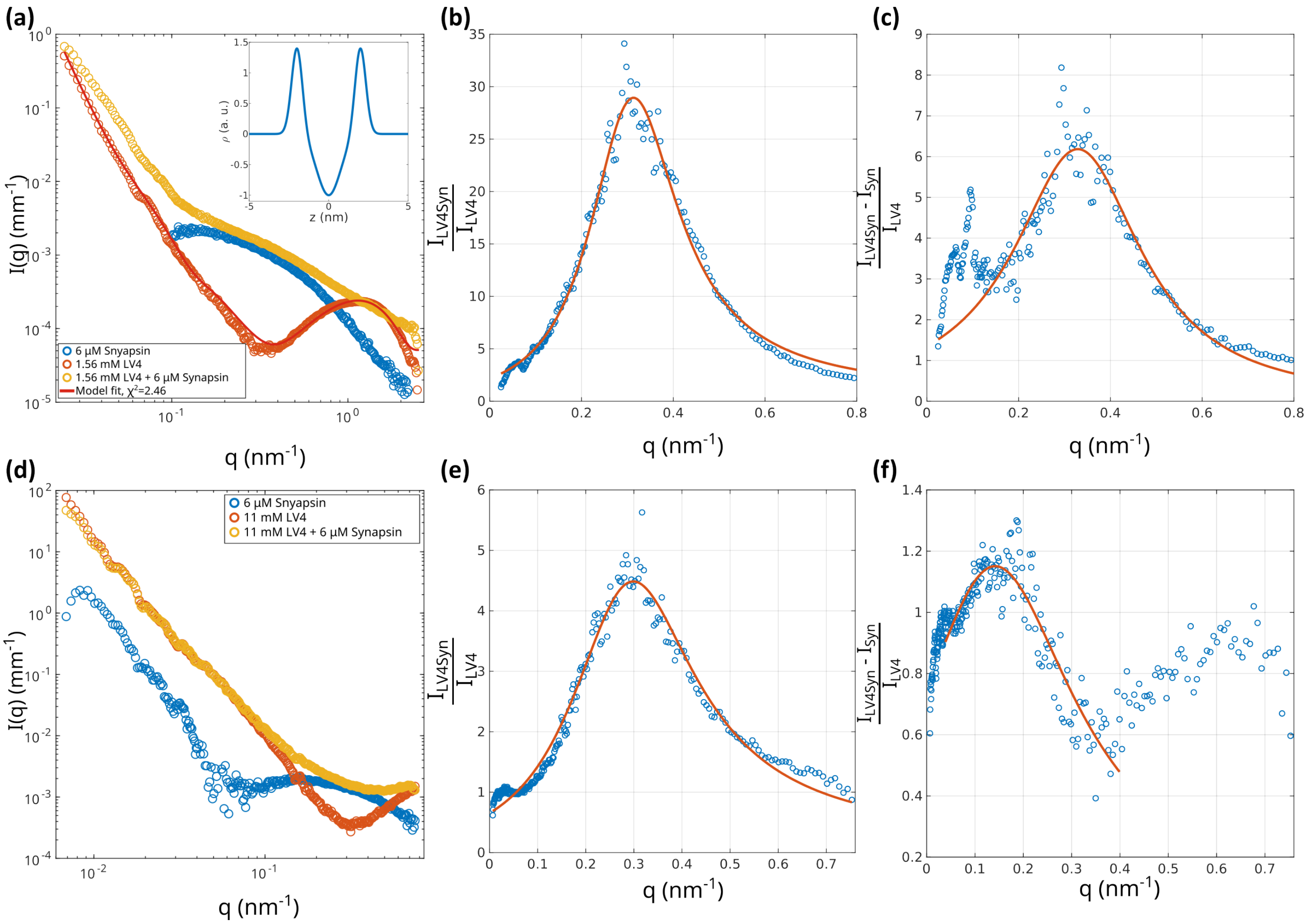

Figure S4: Scattered intensities and corresponding structure factor curves for measurements on LV4-synapsin condensates. (a) Scattering curves of LV4-synapsin condensates at concentrations of 6 μM synapsin and 1.56 mM LV4 (P/L=1:260) measured at a detector distance of 3 m. The acquisition time of the pure synapsin sample was 0.1 s, of the pure LV4 sample 0.5 s and of the condensate sample 0.2 s. The pure LV4 sample was measured at a concentration of 5 mM and the intensity was scaled to fit the intensity of a 1.56 mM LV4 sample. (b) Structure factor of the LV4-synapsin condensates calculated from the scattering curves in (a). The intensity of pure synapsin is not subtracted form the intensity of the condensates. (c) Structure factor of the LV4-synapsin condensates calculated from the scattering curves in (a). In contrast to (b), the intensity of pure synapsin is subtracted before division by the intensity of SVs. (e) Scattering curves of LV4-synapsin condensates at concentrations of 6 μM synapsin and 11 mM LV4 (P/L=1:1833) at a detector distance of 10 m. Even though there is almost no indication of vesicle clustering in the low q-range, which is attributed to the excess of free lipid vesicles at such high lipid concentrations, the signal of the fraction of condensates is visible in the high q-range. This is also visible in the resulting structure factors, which are shown without (e) and with (f) the subtraction of synapsin. Even though an excess of synapsin is not reasonable for such high lipid concentrations, we show this curve for completeness. All samples were measured at an acquisition time of 0.1 s. (e) Structure factor of the LV4-synapsin condensates calculated form the scattering curves in (d). (f) Structure factor obtained from the curves in (d), in contrast to (e), the pure synapsin intensity is subtracted before division. A skewed Cauchy-Lorentz distribution (see Eq. 3) was fitted to all structure factor curves. The model parameters for these fits are shown in Tab. S6.

SUPPLEMENTAL REFERENCES

1. Komorowski, K., J. Preobraschenski, M. Ganzella, J. Alfken, C. Neuhaus, R. Jahn, and T. Salditt, 2022. Neurotransmitter uptake of synaptic vesicles studied by X-ray diffraction. *European Biophysics Journal* 51:465–482. https://doi.org/10.1007/s00249-022-01609-w.

2. Castorph, S., D. Riedel, L. Arleth, M. Sztucki, R. Jahn, M. Holt, and T. Salditt, 2010. Structure Parameters of Synaptic Vesicles Quantified by Small-Angle X-Ray Scattering. *Biophysical Journal* 98:1200–1208. https://www.sciencedirect.com/science/article/pii/S0006349509060913.

3. Pabst, G., M. Rappolt, H. Amenitsch, and P. Laggner, 2000. Structural information from multilamellar liposomes at full hydration: full q-range fitting with high quality x-ray data. *Phys Rev E* 62:4000–4009.

4. Brzustowicz, M. R., and A. T. Brunger, 2005. X-ray scattering from unilamellar lipid vesicles. *Journal of Applied Crystallography* 38:126–131. https://doi.org/10.1107/S0021889804029206.

5. Komorowski, K., A. Salditt, Y. Xu, H. Yavuz, M. Brennich, R. Jahn, and T. Salditt, 2018. Vesicle Adhesion and Fusion Studied by Small-Angle X-Ray Scattering. *Biophysical Journal* 114:1908–1920. https://www.sciencedirect.com/science/article/pii/S0006349518303412.
